# Genome sequence of the basal haplorrhine primate *Tarsius syrichta* reveals unusual insertions

**DOI:** 10.1038/ncomms12997

**Published:** 2016-10-06

**Authors:** Jürgen Schmitz, Angela Noll, Carsten A. Raabe, Gennady Churakov, Reinhard Voss, Martin Kiefmann, Timofey Rozhdestvensky, Jürgen Brosius, Robert Baertsch, Hiram Clawson, Christian Roos, Aleksey Zimin, Patrick Minx, Michael J. Montague, Richard K. Wilson, Wesley C. Warren

**Affiliations:** 1 Institute of Experimental Pathology, University of Münster, 48149 Münster, Germany; 2 Münster Graduate School of Evolution, University of Münster, 48149 Münster, Germany; 3 Primate Genetics Laboratory, German Primate Center, Leibniz Institute for Primate Research, 37077 Göttingen, Germany; 4 Institute of Evolutionary and Medical Genomics, Brandenburg Medical School (MHB), 16816 Neuruppin, Germany; 5 Institute for Evolution and Biodiversity, University of Münster, 48149 Münster, Germany; 6 Integrated Functional Genomics, Interdisciplinary Center for Clinical Research, University of Münster, 48149 Münster, Germany; 7 Medical Faculty (TRAM), University of Münster, 48149 Münster, Germany; 8 Department of Biomolecular Engineering, University of California, Santa Cruz, California 95064, USA; 9 Institute for Physical Science and Technology, University of Maryland, 3300 Metzerott Rd, Adelphi, Maryland 20783, USA; 10 McDonnell Genome Institute, Washington University School of Medicine, St. Louis, Missouri 63108, USA

## Abstract

Tarsiers are phylogenetically located between the most basal strepsirrhines and the most derived anthropoid primates. While they share morphological features with both groups, they also possess uncommon primate characteristics, rendering their evolutionary history somewhat obscure. To investigate the molecular basis of such attributes, we present here a new genome assembly of the Philippine tarsier (*Tarsius syrichta*), and provide extended analyses of the genome and detailed history of transposable element insertion events. We describe the silencing of *Alu* monomers on the lineage leading to anthropoids, and recognize an unexpected abundance of long terminal repeat-derived and LINE1-mobilized transposed elements (*Tarsius* interspersed elements; TINEs). For the first time in mammals, we identify a complete mitochondrial genome insertion within the nuclear genome, then reveal tarsier-specific, positive gene selection and posit population size changes over time. The genomic resources and analyses presented here will aid efforts to more fully understand the ancient characteristics of primate genomes.

## Abstract (web summary)

Tarsiers occupy a key node between strepsirrhines and anthropoids in the primate phylogeny. Here, Warren and colleagues present the genome of *Tarsius syrichta*, including a survey of transposable elements, an unusual mitochondrial insertion, and evidence for positive gene selection.

## Graphical abstract

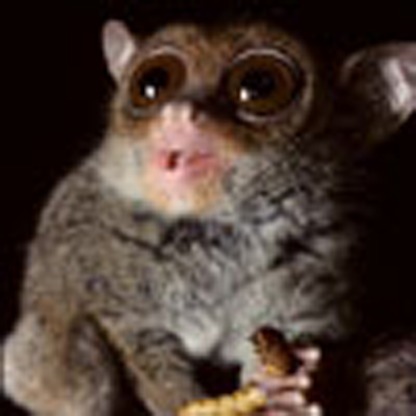

Tarsiers are small nocturnal primates that were, together with their presumed sister family, the extinct Omomyidae, once widespread in Asia, Europe and North America, but today populate only insular Southeast Asia^1^,^2^. The family Tarsiidae contains at least ten extant species that are found in the Indonesian and Malaysian parts of Borneo, the Indonesian islands Sulawesi, Sumatra and Java as well as Greater Mindanao and surrounding islands of the southern Philippines^3^. The Philippine tarsiers were recently ascribed the genus *Carlito*, while the tarsiers of Borneo, Sumatra and Java received the genus name *Cephalopachus*^4^. The genus *Tarsius* was retained for the Sulawesian tarsiers and represents the most speciose of the three genera^5^. Here we adhere to the traditional designation *Tarsius syrichta*.

Tarsiers occupy a key node in primate phylogeny between Strepsirrhini (for example, lemurs, lorises and galagos) and Anthropoidea (that is, monkeys and apes, including humans), and share morphological features with both groups^1^. For instance, like strepsirrhines, tarsiers have an unfused mandibular symphysis, molar teeth with high cups, grooming claws, multiple nipples and a bicornuate uterus. On the other hand, their postorbital closure, absence of a moist rhinarium, hemochorial placenta and retinal fovea are features shared with anthropoids^1^. At the same time, tarsiers are characterized by a number of features uncommon to primates, including dimensions of the craniofacial skeleton and exceptional anatomy associated with various sensory organs, and are the only extant primates lacking deciduous incisors, possibly due to genetic substitutions^6^. Compared with all other mammals, tarsiers have the longest gestation period (178 days) relative to body size.

Tarsiers possess the greatest number of chromosomes compared with other primates^7^. While there are no chromosome painting data available for tarsiers, Tarsiidae exhibit extensive inter-generic diversity in terms of chromosome number, given that members of *Tarsius* possess 46 chromosomes and members of *Carlito* and *Cephalopachus* possess 80 chromosomes^4^. Their evolutionary history, as derived from both the fossil record and molecular characters, remained obscure, especially, as these characters supported all extremes of possible positions in the early divergence of primates: as the first split of primates^8^, in a close relationship to strepsirrhines (that is, prosimian monophyly)^9^, or in a closer relationship to anthropoids (that is, haplorrhine monophyly)^10^,^11^. Past molecular reconstructions mistakenly placed tarsiers on a common branch with strepsirrhines^12^, while most recent evidence supports haplorrhine monophyly, which places tarsiers as the sister taxon to anthropoids to the exclusion of all strepsirrhines^10^,^11^,^13^. Recently, unique ‘molecular fossils' (that is, mobile element insertions) from human and tarsier genomes demonstrated—without conflict—the early Eocene activity of retroposons within the common ancestor of all haplorrhines^14^. Dating posits that within Tarsiidae crown tarsiers shared their most recent common ancestor in the middle Miocene at the latest^5^.

Tarsiers are arboreal primates characterized by large eyes and ears along with long hind legs adapted for vertical leaping^15^. These anatomical specializations allow tarsiers to effectively detect and pounce on prey items. In fact, as the world's most carnivorous primates, tarsiers have extremely large hands and feet compared with their body size^1^,^2^. While tarsiers are generally insectivorous throughout their geographic range, some were observed feeding on lizards and small birds^16^. This dietary regime requires highly specialized optic and auditory adaptations for detecting insect motion and capturing cryptic prey. For example, tarsier eyes comprise a mass that is nearly twice the mass of their brain^17^, and the morphology of the skull reflects the modifications necessary to support a pair of hypertrophic eyes. In contrast to most nocturnal mammals, tarsiers lack a tapetum lucidum, and in contrast to most nocturnal primates, they possess dichromatic colour vision^18^. The primary visual cortex (V1) of the tarsier brain occupies 21% of the neocortex^19^, the largest proportion among all primates, and this neural architecture is likely important for processing detailed vision in dim light^20^. Interestingly, the layered neural morphology of V1 displays a pronounced differentiation pattern that is most similar to that seen in diurnal callitrichids (for example, marmosets and tamarins), which also rely on insects as a primary nutritional resource^21^,^22^. The distinctive pattern of V1 in tarsiers is in fact different from night monkeys, the only nocturnal anthropoids, and strepsirrhines, yet comparable to the pattern found in homologous brain regions of other visual predators, such as the optic tectum in birds^16^. Here, we describe an improved genome assembly that highlights the distinguishing features of genome organization.

Among primate genomes, an obvious novelty is the approximately one million primate-specific *Alu* retrotransposon insertions that are spread almost randomly over all chromosomes. These elements not only expanded the genomic space by >10%, but they also reshaped genomic structure, notably by influencing gene expression or by providing novel protein-coding cassette exons. They are even responsible for various disease phenotypes due to insertion mutagenesis, splicing interference or recombination^23^. Interestingly, most insertions and subsequent evolutionary changes were established after the earliest primate split of strepsirrhines, around 63 million years ago (Myr ago), which clearly predates the evolution of anthropoids, around 40 Myr ago^24^ (for dating see Goodman *et al*.^10^). With tarsiers emerging about 55 Myr ago, the formerly inaccessible period of 15 million years is now available for analysis with this improved genome assembly (for an alternative dating of primate divergences see Perelman *et al*.^13^). Importantly, since it provides the key to the most retrotranspositionally active time period in primate evolution, another goal was to better understand the mechanisms and frequencies of transposon insertion events. In conjunction with the central phylogenetic position of tarsiers in the primate tree most of the critical changes in transposons took place around the divergence point of tarsiers on the lineage leading to—and shaping the largest part of—the human genome. Therefore, a comparative and evolutionary assessment of the transposable element landscape, which comprises at least 44% of the tarsier genome, is central to our investigation, which we complement with analyses of another class of transferred sequences, namely mitochondrial nuclear insertions (numts). Our additional analyses of positive selection, especially for genes associated with the unique biology of tarsiers, as well as a derived historical analysis of the ancient effective population size of tarsiers, provides an impetus for using this updated tarsier genome to explore primate evolution and biology, including both shared and novel aspects within the human lineage.

## Results

### Assembly

We sequenced a female tarsier to 44 × coverage using ABI3730 and Illumina technologies. The combined sequence reads were assembled using default parameter settings within MaSuRCA^25^. The scaffold-level assembly, referred to as tarSyr2.0.1, is 3.4 Gb in size with an N50 contig and scaffold length of 38 kb and 401 kb, respectively. This improved assembly is 13-fold greater in N50 contig length and adds 280 Mb of new sequence not found in the previous low coverage tarSyr1.0 version. Tarsier assembly contig metrics were comparable to previous non-human primate assemblies however, scaffold length is on the lower end of the observed range (Supplementary Table 1). We predicted 20,820 protein-coding genes that, when aligned against a conserved eukaryotic gene set, demonstrated complete coverage for 97.6% (447/458) of core genes^26^ and alignments of predicted tarsier proteins to RefSeq proteins from other primate species (*n*=14,055) demonstrated an average coverage of 90% with an average identity of 82%. We also provide annotation of the non-protein coding RNAs (npcRNAs), specifically snoRNAs and miRNAs (Supplementary Information; Supplementary Fig. 1; Supplementary Data 1 and 2). We detected 223 snoRNAs (plus 150 isoforms/variants) in the tarsier genome based on the identification of human homologues^27^ (Supplementary Table 2; Supplementary Data 1). Our list of expressed snoRNA and miRNAs show significant similarities to human, including the proximate mechanisms by which protein complexes are guided to specific positions in other RNA entities^28^. Putative alternative pathways of miRNA generation are also conserved between tarsier and human (Supplementary Table 3; Supplementary Data 2). In summary, we contend the tarsier assembly is of sufficient quality to enable all analyses described in this study.

### Repeat elements

Three different strategies were used to examine the transposon landscape of the newly assembled tarsier genome: (1) a classical RepeatMasker analysis of the entire genome, (2) development and subsequent screen of a pairwise genome alignment of *Homo sapiens/T. syrichta* (hg19/tarSyr2.0.1) and (3) a transposition in transposition (TinT) analysis to delineate the relative activity periods of transposed elements over time. This enabled us to differentiate between elements that were active before the branching of tarsiers (present in human and tarsier) and those that are tarsier-specific compared with anthropoid primates (active after branching point). Below we present an overall summary of repeat elements specific to the tarsier genome and discuss their emergence within an evolutionary framework (Table 1; see Supplementary Table 4 for family metrics).

**Figure 1:**
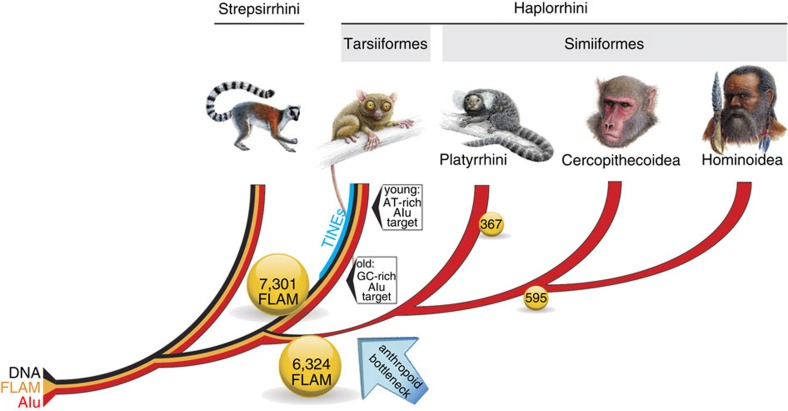
The *Alu* and DNA element histories of the main primate lineages. The key position of the tarsier between Strepsirrhini and Simiiformes (Anthropoidea) is depicted. DNA transposons (DNA; black line) and fossil left *Alu* monomers (FLAMs; orange line) were active at the origin of primates but became inactive sometime after tarsiers diverged from the anthropoid lineage, possibly during an intensive anthropoid population bottleneck. *Alu* elements were active in all primate lineages (red line). Young tarsier *Alu*J elements (with perfect target site duplications—TSDs) show a preference for more AT-rich target sequences compared with older *Alu*J elements (with diverged TSDs), which accumulated in more GC-rich regions. TINE elements evolved on the tarsier lineage (blue line). FLAMs (7,301) were detected on the tarsier branch and 6,324 FLAMs inserted in the common ancestor of anthropoids. Afterwards, the activity of FLAMs within anthropoid lineages was significantly reduced. Only 367 elements were detected in the marmoset (Platyrrhini) and 595 elements were estimated for the lineages leading to human. Drawings of animals are provided by Jon Baldur Hlioberg. Drawings for Tarsiiformes and Hominoidea have been reproduced from Hartig *et al*.^14^ with permission.

To explore the *Alu* element history we sought to examine two key features of *Alu* SINE (short interspersed element) history; first, their enrichment within potential gene-rich (high GC-content) regions in an element age-dependent manner and second, their lineage-specific monomer and dimer element histories. While human LINEs (long interspersed elements) are strongly biased towards AT-rich regions, SINEs are typically found within GC-rich regions of the genome^29^. Furthermore, in human, *Alu* SINEs display an age-dependent accumulation in AT- or GC-rich regions; younger *Alu*s are preferably located in AT-rich regions, while the older ones are slightly enriched within the more GC-rich parts of the genome^29^. With the tarsier genome, and its comparison to human, we provide a distant reference point for age-dependent *Alu* insertions. When we examined old (>30% divergent target site duplications (TSDs)) and young (identical TSDs) *Alu*J elements and calculated the relative GC-content within 50-kb windows surrounding each element, we observed a significant enrichment of young *Alu*s within AT-richer regions compared with older *Alu*s (F-statistics, *P*<0.05; Fig. 1). We identified the opposite distribution for young and old LINEs (F-statistics, *P*<0.05), with the latter represented in more AT-rich regions (Fig. 1). Furthermore, we observed a significantly different distribution for *Alu*s versus LINEs (F-statistics, *P*<0.05), the latter were enriched in more AT-rich regions. This tarsier result demonstrates a more general primate-wide phenomenon of *Alu* history^29^,^30^. It is possible that highly active *Alu* elements are selectively ‘sorted out' from the functional GC-rich areas of the genome.

Given the ample evidence that the parallel activity of different *Alu* elements is restricted in time, it is likely that *Alu* dimers replaced monomeric elements during the early divergences of primates^31^. Our analysis, based on pairwise alignments (human/tarsier—hg19/tarSyr2.0.1; human/marmoset—hg19/calJac3), revealed 7,301 tarsier-specific *Alu* monomers (especially Fossil Left *Alu* Monomers—FLAMs) that were inserted in the lineage leading to tarsiers, compared with 6,324 that were fixed in the lineage leading to anthropoids and 595 within the subsequent lineage leading to human; yet, we detected only 367 New World monkey-specific monomers (Fig. 1). The extreme reduction (by >90%) in the lineage-specific accumulation of monomeric *Alu*s is difficult to explain by relatively rare cases of tailless retropseudogenes (internal priming of reverse transcription)^32^ or classical recombination processes^33^. This implies that *Alu* monomers remained active after the divergence of tarsiers from the haplorrhine stem (Fig. 1) and for a limited time on the lineage leading to anthropoids, where they then became nearly extinct. However, within anthropoids, the lower number of detectable lineage-specific *Alu* monomers indicates inactivation with rather coincidental generation of only relatively few monomer-like tailless retropseudogenes (internal *Alu* priming generates monomer-like sequences^34^) or even recombination^33^. However, the high number of *Alu* monomers at the haplorrhine/anthropoid split correlates with a burst of LINE1 elements that can co-mobilize among other elements such as monomeric *Alu* SINEs (see Fig. 1 of Pace and Feschotte^35^ for example).

In primates, the last observed intense activity of DNA transposons occurred on the lineage leading to anthropoids^35^. During that time, the tarsier lineage had already diverged, and we therefore hypothesized that it might reveal different DNA transposon activity patterns. The reported pattern of mammalian DNA transposon activities comprised 284,000 elements inserted in the common mammalian ancestor, with 74,000 elements in the common primate ancestor, and 23,000 in the ancestor of anthropoid primates. In an analysis of tarsier transposon insertions using the Genome Presence/Absence Compiler (GPAC), which screens for clear cases of presence or absence^34^, we determined from 29,966 full-length DNA transposons with clear presence/absence states (allowing for 20 nucleotide truncations; from a total of 126,481 full-length DNA transposons) that 87% (*N*=26,079) inserted in the ancestral lineage leading to Scandentia plus primates, 2.8% (*N*=849) inserted in the common ancestor of primates, 0.3% (*N*=91) in the haplorrhine ancestor, reflecting the short branch of haplorrhines, and 10% (*N*=2,930) in the ancestral anthropoid lineage. Interestingly, only a few insertions (∼0.05%; *N*=17) were identified after the divergence of New World monkeys from the lineage leading to human. The hg19/tarSyr2.0.1 pairwise alignment revealed that 761 *Charlie* (hAT elements), 196 *Tigger* (Tc1/mariner elements), and 117 *Mariner* (Tc1/mariner elements) DNA transposons were restricted to tarsiers, which we consider a strong indication of specific activities on the branch leading to Tarsiiformes (Supplementary Fig. 2).

**Figure 2:**
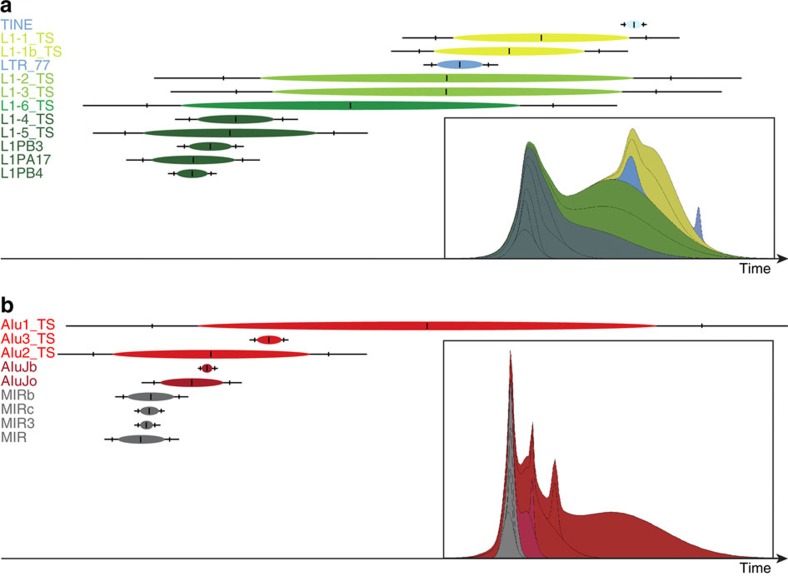
Transposition in transposition (TinT) patterns of TINEs and SINEs over time. (**a**) Old LINE (L1) elements are displayed in dark green, younger L1s in light green. The TINEs and their proposed LTR_77_TS source gene are depicted in blue. The period of maximal activity is indicated as a central vertical line within the coloured ovals. The flanking vertical lines represent the 75th percentile of the activity range; the terminal lines show the 99th percentile range of activity. The timescale runs from left to right. (**b**) Transposition in Transposition (TinT) pattern of SINE elements, including mammalian-wide interspersed repeats (MIRs; grey), the primate-specific *Alu*Jb and *Alu*Jo (dark red), and the tarsier-specific *Alu*1-3_TS elements (red). The period of maximal activity is indicated as a central vertical line in the coloured ovals. The flanking vertical lines represent the activity within the 75th percentile range; the terminal lines represent the 99th percentile intervals. The corresponding cumulative likelihoods of element family activity are given as thumbnails in the insets.

In addition to LINEs, SINEs and DNA transposons, we also observed and examined TINEs (tarsier interspersed elements), repetitive elements specific to the tarsier genome. TINEs accumulated in high copy number (see Supplementary Fig. 3 for molecular overview) and were probably derived (that is, transcribed) from a tarsier-specific long terminal repeat (LTR77_TS) that was retropositionally silenced for a long time (there are no overlapping activity patterns with LTR77_TS and TINEs; see Fig. 2). We found 29,041 tarsier TINE1/2 copies (26,938 full length), previously described only as SINE-like repeats (www.girinst.org). Some investigators might suggest these are not necessarily SINE elements but instead ought to be classified as short retrosequences. We also identified 1,668 transposed elements representing another tarsier-specific repeat, which we refer to as TINE3s. These elements were likely derived from an inactivated MER83B-like LTR element that was also retropositionally inactivated for a long time, analogously to the mechanism suggested for TINE1/2-like transcription (Supplementary Fig. 2). The TINE1/2 and TINE3 elements on average exhibit 88 or 89% sequence similarity to their consensus sequences, respectively, indicating that TINEs were recently active, which is also supported by their TinT patterns (see below and Fig. 2a).

TinT analyses reflect the relative activities of transposed elements over time by comparing the numbers of different elements that integrated into one another^36^. Elements that harbour integrations of many different element families but are not detected inside of other elements are considered to be older and mostly inactive. On the other hand, elements inserted into many other elements but not occupied by them represent younger elements. We found that the LTR77_TS and tarsier-specific TINEs show recent activity that overlaps with the activity patterns of two tarsier-specific LINEs that might provide the necessary enzymatic machinery for TINE mobilization; this was further supported by overlapping TinT patterns (Fig. 2a). We also measured the activity ranges of tarsier SINEs, including some tarsier-specific *Alu*s (for example, *Alu*1_TS), and determined that activity is both ancient and most likely ongoing (Fig. 2b).

**Figure 3:**
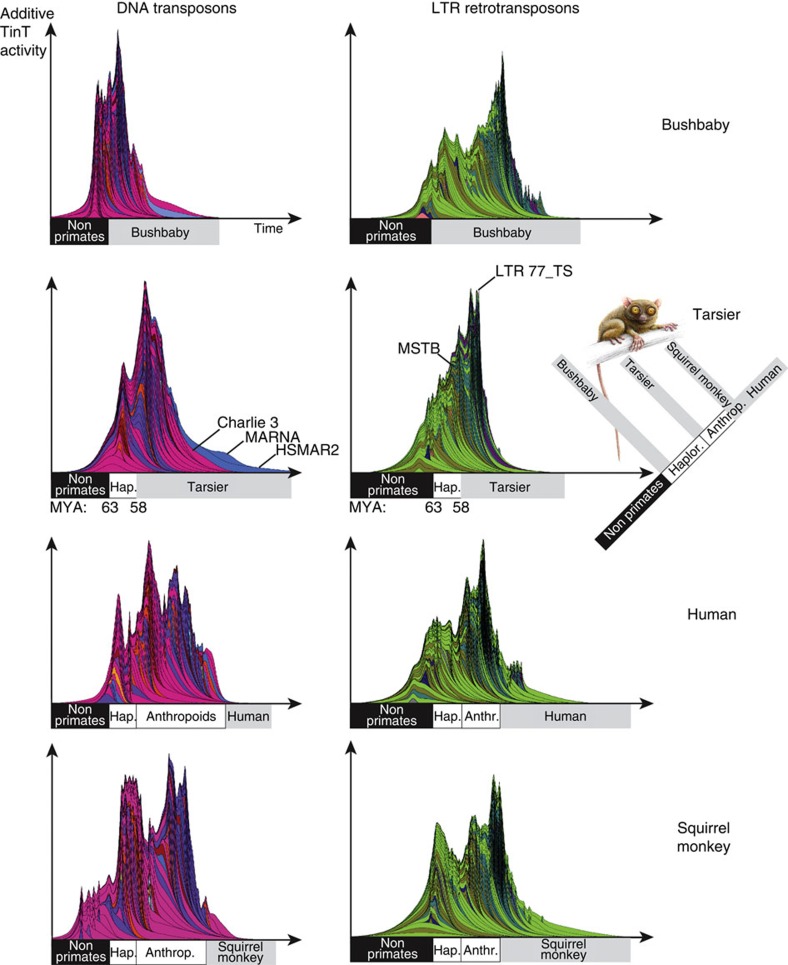
Cumulative likelihood of activity for DNA transposons and LTR retrotransposons. Cumulative TinTs are shown for bushbaby (Strepsirrhini *Otolemur garnettii*), tarsier (*Tarsius syrichta*), human (*Homo sapiens*), and squirrel monkey (New World monkey *Saimiri boliviensis*) genomes. Patterns on the left show TinT genome scans for DNA transposons (red/blue; different layers show different element families), while those on the right depict TinT genome scans for LTR retrotransposons (green; different layers show different element families). The cumulative non-primate, prosimian-specific and tarsier-specific transposons are indicated below each plot. The bushbaby TinTs represent the earliest divergence of primates. Their DNA transposon cumulative TinT pattern is similar to that of the tarsier. However, the LTR retrotransposons show a somewhat more heterogeneous pattern indicating changes in the population size or changes in LTR activity. In tarsier, the dominating DNA transposons are the Charlie3, MARNA, and HSMAR2 elements, which display recent activity in the tarsier. The ERV MSTB retroposons represented the most informative phylogenetically diagnostic insertions to clearly assign the common ancestor of Haplorrhini, including tarsiers and anthropoids, to a natural phylogenetic group^14^. The LTR77_TS elements were relevant for the transcription and genomic propagation of nearly 30,000 TINE retropseudogenes. The New World, squirrel monkey presents a rugged additive TinT pattern similar to that of human. However, the early anthropoid divergence and distribution of individual New World monkey elements also demand some obvious deviations from the human pattern. Timescales for each pattern run from left to right. The phylogenetic tree to the right indicates the different areas of the cumulative TinT profiles, separated into (1) non-primates, (2) prosimians and (3) tarsier or human (see also timescale for the first primate split at 63 Mya and the divergence of tarsier at 58 Myr ago). For the principles of the cumulative presentation of TinT patterns see Fig. 2. Drawings of animals are provided by Jon Baldur Hlioberg. Drawing of Tarsiiformes has been reproduced from Hartig *et al*.^14^ with permission.

Cumulative TinTs represent the accumulated retroposon fixation probabilities over time^37^ and therefore provide more refined information about the historical patterns of these unique genome-shaping events. We conducted such analyses for the genomes of tarsier and human in comparison to bushbaby and squirrel monkey, which identify the proportion of the tarsier TEs that inserted into other TEs arising from non-primates, the proportion that was active in prosimians, and the proportion that are tarsier- or human-specific (Fig. 3). Our analyses revealed that prosimians and anthropoids have different cumulative TE insertion patterns (Fig. 3). In particular, the observed bottleneck in tarsier populations that persisted over a long period of time resulted in a very compact cumulative pattern. Historical changes in population structures on the lineage leading to human are reflected by a more heterogeneous cumulative TinT pattern (that is, interrupted by many valleys within the graphical representation), which might reflect extended periods with larger effective populations sizes and comparatively lower rates of transposon fixation events (Fig. 3). Although one might expect a greater degree of similarity between the tarsier and human patterns for the homologous, ancient elements (that is, the non-primate/non-prosimian area of the TinT), this zone differs due to more recent species-specific elements, each integrating independently into these silent elements. These activities—in species as distant as tarsier and human—reshape the historical (that is, shared) patterns in different ways, with the general shape of the patterns—the peaks and valleys—providing estimates about the occurrence of different insertion activities.

### Tarsier-specific nuclear mitochondrial DNA

**Figure 4:**
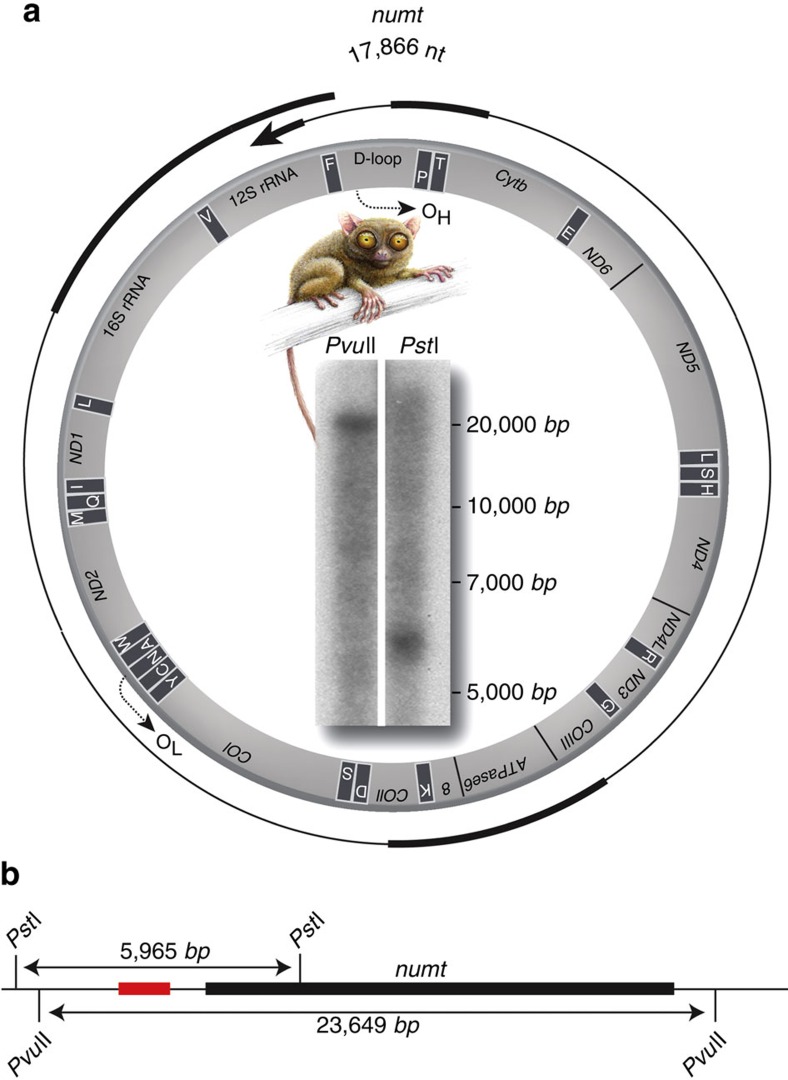
A gene map of the tarsier-specific nuclear full-length mitochondrial (numt) DNA insertion. (**a**) The 17,866 nt numt covers the complete mitochondrial genome and also overlaps in the 12S rRNA/D-loop region. The genome organization of the tarsier mtDNA is displayed in the central ring in grey. A Southern blot of the numt is presented inside the central ring. (**b**) The restriction sites (*Pst*I and *Pvu*II digestions), probe location (red bar), and corresponding lengths of the probes (5,965 bp and 23,649 bp) are denoted at the bottom (details are presented as Supplementary Fig. 6). The tarsiiform drawing was reproduced from Hartig *et al*.^14^ with permission.

In addition to the retro- and DNA element transpositions described above, the tarsier genome harbours various randomly integrated mitochondrial fragments. Such nuclear mitochondrial DNA insertions (numts) usually remained silent in their new surroundings, and today we usually observe only their fragmented traces however, the newly assembled tarsier genome contained a novel numt. Based on our pairwise genome alignments of *H. sapiens/T. syrichta* (hg19/tarSyr2.0.1), all tarsier-specific sequences, including their flanking regions (50 nt), were extracted and screened for tarsier-specific numt DNA insertions. Only two of the tarsier-specific regions contained mitochondrial DNA. The first region comprised ∼3 kb of the mitochondrial rRNA genes and displayed 90% similarity to the homologous mitochondrial sequence. Interestingly, the second insertion spanned the entire mitochondrial genome with further overlaps of the 12S rRNA and D-loop regions (Fig. 4) and exhibited 88% sequence similarity to its mitochondrial counterpart. This example represents the first report of a complete mtDNA genome integration into a mammalian nucleus. It is likely the result of recombination between pre-existing numts. The authenticity of this insertion was experimentally reconfirmed by sequencing the PCR products derived from both flanks and by Southern blot analysis (Fig. 4; Supplementary Data 3; Supplementary Fig. 4). To deduce a complete and comparative picture of primate-wide numt insertion patterns, we also screened for such elements in other primates (Supplementary Tables 5 and 6). Our analysis differentiated lineage-specific from shared numt elements as a result of inspecting pairwise and multi-way primate genome alignments with GPAC^34^. The resulting insertion patterns indicated a continuous process of genomic insertions of random mitochondrial fragments during primate evolution with an increasing accumulation on internal and terminal anthropoid branches (Supplementary Fig. 5).

### Positive selection in the tarsier

Many of the unusual anatomical and physiological features of Tarsiidae remain obscure, yet in a genome-wide screen for changes associated with specific biological features, such as night vision and unique locomotion, we did detect potential relationships linking protein-coding changes with such characteristics. Changes in amino acids can result in positive Darwinian selection if they acquire adaptive value. We used the codeml package as implemented in PAML^38^ to detect branch-specific positive selection, based on the branch-site test comparing the H0 (not allowing positive selection) and H1 (allowing positive selection) models in a likelihood ratio test. A common subsequent analysis is the ranking of associations between a set of known disease-related genes and a set of genes under positive selection. We detected a total of 192 genes under positive selection (Supplementary Table 7) that were then tested for disease association (Supplementary Table 8) and canonical pathway enrichment (Supplementary Table 9). A total of 47 diseases were output with *P* values <0.05 and FDR <0.05 (Benjamini and Hochberg corrected^39^) using ToppGene^40^ (Supplementary Table 8). Eight positively selected genes within the tarsier lineage were implicated in several diseases associated with eye development and visual disorders (*BBS2*, *BFSP2*, *IDUA*, *IL1A*, *IMPG1*, *RGR*, *SRD5A3* and *TMC8*). Visual deficiencies accounted for 25% of the significantly enriched diseases. Kahrizi syndrome, for example, is caused by a homozygous truncating mutation in the *SRD5A3* gene and results in abnormal eye development, including the early onset of cataracts and coloboma (that is, holes) within the iris^41^. In addition, musculoskeletal disorders accounted for 23% of the significantly enriched diseases (Supplementary Table 8). In one example, gene mutations in the sarcomeric protein telethonin (*TCAP*) cause calf hypertrophy and limb-girdle muscular dystrophy, which results in proximal atrophy in the upper limbs and proximal and distal atrophy in the lower limbs^42^. To further refine our biological inference of tarsier genes under positive selection, we also examined 161 gene sequences orthologous to human for significant associations with curated pathway databases^43^ (Supplementary Table 9). Overall we find statistical support for other networks, such as two positively selected genes (*RGCC* and *WNT11*) that are associated with the negative regulation of *FGF* production (Supplementary Table 9; for species included in the analysis of positive selection see Supplementary Table 10).

### Tarsier population history

**Figure 5:**
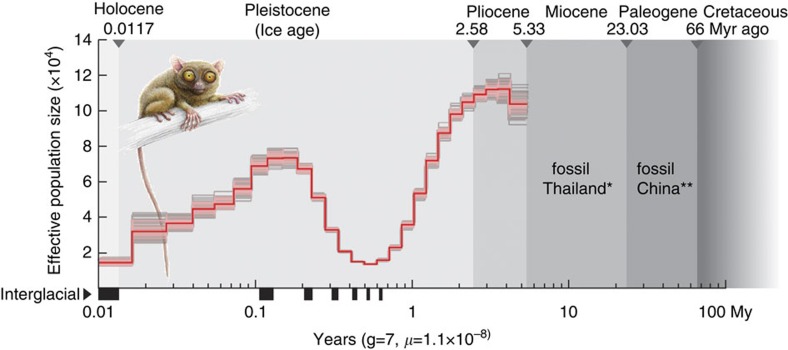
Historical effective population sizes of *T. syrichta.* *N*_e_ was derived by the pairwise sequential Markovian coalescent model (PSMC)^44^. The *x* axis gives a log scale of the time in years (adapted to the major glacial epochs), applying a genome mutation rate of *μ*=1.1e−8 per site and generation time of 7 years^66^. The bold red line shows the effective population size through time. The thin pink and grey lines represent 100 rounds of bootstrapped sequences. The interglacial epochs are indicated with black bars on the *x* axis. *A fossil tarsier from the middle Miocene of Thailand^47^; **a fossil tarsier from the Eocene of China^46^. The drawing of tarsier has been reproduced from Hartig *et al*.^14^ with permission.

Demographic history and population fluctuation were undoubtedly influenced by climate variability throughout the Miocene, Pliocene and Pleistocene. We used the diploid genomic sequence and the pairwise sequential Markovian coalescent (PSMC) model^44^ to infer historical fluctuations in the effective population size of *T. syrichta* (Fig. 5). The conceivable demographic history and population fluctuation were likely significantly influenced by the Pleistocene glacial and interglacial variations in temperature during the last 2.58-million years. The latest glacial period (∼10,000 years ago) corresponds with the current low-level plateau in the population size, as shown during the Holocene (Fig. 5; *N*_e_ ∼20,000; for the Pleistocene and Holocene glacial epochs^45^), while the many times higher effective population size during the Pliocene was perhaps related to alternating glacial and interglacial periods. The decline in the effective population size (*N*_e_ ∼20,000) during the Pleistocene, about 0.6–1 Myr ago, possibly reflects the influence of climatic changes associated with the early Pleistocene, about 2.58 Myr ago. However, this putative bottleneck is not apparent from the tarsier-specific analysis of the cumulative TinTs (Fig. 3). Even with the oldest fossil evidence for tarsier dated to the middle Eocene epoch (∼40 Myr ago) in China^46^,^47^, the relatively sparse fossil record and the estimated period for crown tarsier speciation (about 22 Myr ago 48) place limits on the PSMC model and historical reconstruction. We also note that the PSMC model only derives a comparatively rough approximation of the historical course of population size; it varies with changing generation time, mutation rate estimates, and assembly quality. Nonetheless, a detailed protocol of the PSMC reconstruction is provided in the Supplementary Information.

## Discussion

Due to its unique phylogenetic placement among primates, as sister taxa to anthropoids, tarsiers are integral for understanding primate genome evolution, and these first analyses of an improved tarsier genome assembly reveal novel insights into ancestral features of our own genomes. More specifically in this study, we better define the complex interrelationships among sustainment by adaptation (for example, positively selected genes), genome architectural changes (for example, by transposed elements), and epoch changes that possibly altered tarsier populations in pre- and post-modern times (for example, probable root in the Miocene/Eocene). We first focused on a detailed analysis of the genomic landscape of transposed elements (including subfamilies), which putatively include unique expansion events, given the expanded size of the tarsier genome relative to other primates^49^. Previous data, lacking information from tarsiers, indicated that DNA transposons were active at the initial split in primates but remained silent in all anthropoids^35^. Results from our present analysis clearly indicate activity in the haplorrhine lineage leading to tarsiers. Based on this information and multiple pairwise genome alignments, we posit a narrower estimated time frame for transposon inactivation in primates, with its occurrence in the lineage leading to anthropoids after the divergence of *Tarsius* (between 58 and 40 Myr ago^10^). A similar inactivation pattern was observed for monomeric *Alu* elements, strongly indicating that *Alu* dimers and monomers were simultaneously active for millions of years until monomers became silent only in the lineage leading to anthropoids after the divergence of tarsier. Different processes subsequently produced monomer-like, lineage-specific elements in anthropoids^34^.

The cumulative activity pattern of TEs in the lineage leading to *Tarsius* also indicated a very different individual element fixation history compared with human. The tarsier pattern was less heterogeneous, which putatively resulted from comparatively higher rates of transposon fixation events due to extreme bottlenecks in tarsier populations that persisted over a long period of time compared with the human lineage (Fig. 3). Primates are unique among therian mammals with the highest activity of the autonomous LINE1 retrotranspositional machinery. This is illustrated by the extremely high retrotranspositional activity of LINE1 co-retrotransposed non-autonomous *Alu* SINEs^29^ (for the tarsier see Fig. 2b). LINE1 retrotransposons also co-retrotranspose more or less exclusively therian-specific retropseudogenes. Most of them are equipped, similar to LINEs, with an A-tail that increases the affinity to the LINE1 machinery^50^ (for example, mRNAs but also many other polyadenylated transcripts such as TINEs). In the tarsier, an unusually high number of TINEs were derived from an ancient and otherwise defective LTR with internal polyadenylation signals and a functional RNA polymerase II promoter. We detected tens of thousands of such tarsier-specific TINE copies mobilized by the LINE1 machinery and distributed genome-wide (Fig. 1; Supplementary Fig. 3). In addition to delineating the process of activation and propagation of these elements, we also found thousands of novel TINE elements, presumably derivatives of LINE1-mobilized MER83B partial transcripts.

Human LINE1 elements were reported to have a different distribution pattern than *Alu* SINEs, in that LINEs accumulated in AT-rich intergenic regions and *Alu* SINEs in more GC-rich, gene-rich genomic environments^29^. More detailed analysis showed however that older *Alu* SINE elements accumulated in more GC-rich regions while younger elements were relegated to AT-rich regions. The tarsier genome provided an opportunity to explore these patterns at greater phylogenetic depths, and our observations suggest the process is probably an ancestral feature in most primates.

The transfer of mitochondrial DNA into the nuclear genome (numts) is common but varies in scope among species^51^. In addition to insertions of transposed elements, we also examined the insertions of specific mitochondrial sequences in tarsiers, and to our knowledge, describe for the first time in mammals the integration of a complete mitochondrial genome into the nuclear genome (Fig. 4). In human, numts were estimated to have continuously occurred over a 58-million year period, mostly as a result of new events and not primarily through duplication of existing numts^52^. We contend this unique tarsier event offers a new perspective in comparing the evolutionary changes between the rapidly changing mitochondrial DNA and slowly changing nuclear sequences, both originating from the same source but evolving under very different selective constraints. Moreover, analysing the frequent phenomenon of nucleotide compositional changes in mitochondrial sequences and the duplication incidence of this intact mitochondria genome could provide new theories related to selection processes in the nuclear genome.

The evolution of the tarsier genome as a result of shifting repetitive elements and positive selection on the protein-coding regions represent some of the footprints of natural selection. Tarsiers are nocturnal insectivores, characterized as the most carnivorous primate, with morphological specializations of the visual and motor systems that render the species well adapted for leaping and catching prey. Correspondingly, we detected tarsier-specific positive selection in several genes of the musculoskeletal and visual systems that are presumptively essential for this unique biology, relative to other primates. Interestingly, *FGF12*, a member of the fibroblast growth factor family of genes, and genes that regulate *FGF* cellular levels (*RGCC* and *WNT11*) are all under selection pressure in the tarsier lineage. This FGF family has been implicated in supporting chondrogenesis and osteogenesis in a broad scope of studies^53^. If these gene pathways within a broader network are under evolutionary selection in tarsiers relative to other primates, perhaps these genes, as well as other yet unexplained contributors facilitate development of the unique musculature and skeletal system of the tarsier, such as their uncharacteristically large extremities compared with their body size^1^,^2^. Notably, other members of this network, for instance *IL1A*, support a model of general hypertrophy (Supplementary Fig. 6), suggesting putative underlying implications for the unique biology of tarsiers, including increased skeletal muscle mass^54^ to facilitate leaping behaviour. Alternatively, it may be partially responsible for the extreme orbital hypertrophy of tarsiers as an adaptive response for insect capture within a nocturnal environment. In addition, we highlight a collective of eight genes associated with various eye development disorders, such as *BFSP2*, that in null mice display a deterioration of light scatter and lens optical properties^55^, providing further evidence for the adaptive importance of the visual system with regards to tarsier survival. The reported tarsier-specific protein alterations afford opportunities to refine our understanding of their role in differentiating the novel anatomical features of the tarsier; however, further molecular experimentation and more extensive sequencing of tarsier populations is needed.

Tarsiers were once a globally distributed phylogenetic group, but are currently restricted to Asia's Sundaland region. Given the questionable future of various tarsier populations due to continued habitat destruction, their conservation is of upmost importance^56^. This is apparent from our measurement of the effective population size, which is currently depicted as the lowest in tarsier's history (Fig. 5), contrasting remarkably with many mammals, including human^44^. Tarsier's unique position in the primate phylogeny, between the most basal strepsirrhines and the more derived anthropoids, including human, represents an exceptional link to understand and analyse our own evolution and to identify the ancient prerequisites for novel gene formation and disease-causing changes in primates. We hope that the improved tarsier genome assembly will stimulate many follow-up studies of biodiversity and primate genetics, while also drawing deserved attention to this highly unique member of the primate order.

## Methods

### Source material

Muscle and brain tissues from a female Philippine tarsier (*T. syrichta* sensu *Carlito syrichta*) were provided by the Duke University Lemur Center (Durham, NC, USA). Genomic DNA and RNA isolations were performed according to standard protocols.

### Genome assembly and annotation

From this single female DNA source, a series of read types were generated with various sequencing technologies, including 1.82 × whole-genome coverage on ABI3730 instruments (1.7 M plasmid and 600 fosmid end reads), 34 × short-(350 bp fragment size), and 8 × long-insert (8 kb fragment size) paired reads on the Illumina Hiseq2000 instrument. The combined sequence reads were used to generate a *de novo* assembly (Tarsius_syrichta-2.0.1; accession GCA_000164805.2) using the MaSuRCA assembler^25^. The pipeline used for gene annotation of the tarsier assembly is found in the NCBI handbook (http://www.ncbi.nlm.nih.gov/books/NBK169439/).

### snoRNA and miRNA detection

Total RNA was isolated from 100 mg brain tissue using the *mir*Vana miRNA Isolation Kit (Live Technologies GmbH, Frankfurt). The quality of RNA was checked with the Agilent 2100 Bioanalyzer (RNA nano chip). We added the ERCC RNA Spike-In mix for control and extracted the small RNA transcriptome and depleted ribosomal RNA from 5 μg RNA (RiboMinusTM Eukaryote System v2). Efficient depletion was controlled using the Agilent 2100 Bioanalyzer (RNA nano chip). The Ion Total RNA-Seq Kit v2 was used to construct the small RNA library. Sequencing was performed with an Ion Proton sequencer.

To detect potential snoRNAs in the tarsier, we screened for known human snoRNAs (https://www-snorna.biotoul.fr/). We utilized local blastN to screen our entire tarsier brain small RNA transcriptome (1,338 extracted candidates) with 939 annotated (UCSC: https://genome.ucsc.edu/cgi-bin/hgTables) and 2,469 predicted human pre-microRNAs^9^.

### Repeat elements of the tarsier genome

A local version of RepeatMasker was used to derive the transposon landscape of the *T. syrichta* genome (tarSyr2.0.1; http://www.repeatmasker.org/RMDownload.html; giri RepeatMasker library http://www.girinst.org/). For the comparative TinT analyses, the human RepeatMasker report was downloaded from UCSC (http://hgdownload.cse.ucsc.edu/goldenPath/hg38/bigZips/hg38.fa.out.gz). The genome-size was calculated by counting all ‘non-N' characters. From the tarsier RepeatMasker report, we extracted all repeat coordinates and grouped them by element names. For every element group, we calculated: (1) the number of full-length forms (±10 nt truncations allowed); (2) the fraction of nucleotides occupied by individual element groups in the genome; and (3) the number of all elements. We also calculated the number of tarsier-specific elements using our human/tarsier pairwise alignment, and counted all elements occupying >70% of sequence regions present in tarsier but absent in human. We considered elements that exceeded ten copies to be potentially active. Counts for TINE3 elements were added manually because these elements are currently not represented in the RepeatMasker library.

Using the TinT Java application^36^ we created TinT and cumulative TinT profiles for different elements in tarsier, human, and other primates based on standard parameters. For cumulative TinTs, we merged small element fractions (<5 lineages) and element groups (<1,500 individual elements). To calibrate the timescale for species boundaries, we compared the individual TinT patterns from all available primate genomes and selected overlapping elements of non-mammalian, prosimian, or tarsier-specific origin (Fig. 3).

We used the Philippine tarsier mitochondrial genome (NC_012774.1) for numt insertion analysis. Masking of repetitive elements was conducted with the RepeatMasker tool (http://www.repeatmasker.org/RMDownload.html; see Supplementary Information for settings). To calculate the similarity between numts and their original mitochondrial sequences and between TINE1/2/3 consensus sequences and genomic full-length hits, we calculated the Levenshtein-Distance^57^ by measuring the number of character changes needed to convert one sequence into the other.

The pairwise alignment (*H. sapiens/T. syrichta*-2.0.1) was based on the soft-masked human genome (hg19; http://www.repeatmasker.org/species/hg.html) and was generated as previously described (Supplementary Information). From the hg19/tarSyr2.0.1 pairwise alignment, 422,362 unique tarsier-specific regions were extracted. These regions were filtered for sequences >30 nucleotides (nt) and those corresponding to gaps in the human genome with a maximum of 10-nt extensions in the 5′- and 3′-directions. All unique tarsier-specific regions were extracted with a C++ script (available on request).

From the hg19/tarSyr2.0.1 pairwise alignment, 7,301 unique loci were detected in the tarsier genome containing FLAMs or FRAMs versus 6,919 for the lineage leading to human. The 6,919 human loci were verified for presence/absence in marmoset using the hg19/calJac3 pairwise alignment downloaded from UCSC (http://hgdownload.soe.ucsc.edu/goldenPath/hg19/vsCalJac3/). For marmoset we detected 367 specific monomers. Based on the proportion of the 2,304 detectable monomeric loci in marmoset corresponding to the 6,919 loci in human and the recognition of 198 cases absent in marmoset, we approximate the numbers of *Alu* monomers for the human lineage that diverged from a shared ancestor with New World monkeys (198/2,304) × 6,919=595.11 rounded to 595 cases with remaining 6,324 cases for the ancestor of anthropoids.

The 17-way species genome alignment parameters, including the leading tarsier genome, are described in supplementary methods. During the first analysis steps, pairwise LASTZ alignments^58^ between tarsier and the remaining species were prepared using repeat-masked genome sequences. Each scoring matrix was tuned based on phylogenetic distance and the coverage of the respective genome, joining alignments into chains if close enough. During this step, a kd-tree from the gapless subsections of the alignments was computed. With a dynamic programme, the maximally scoring chains of these subsections were extracted and placed alongside the genomes. To produce an alignment net, gaps were filled with lower-scoring chains (parameters were calculated depending on the genomes used). Finally, all of these pairwise alignments were used to generate the 17-way alignment with MULTIZ^59^. The same procedure was carried out to prepare a 17-way alignment with the identical genomes but with human as the leading species.

To identify novel, tarsier-specific transposed elements, the pre-masked ‘unique tarsier sequences' were used as a library for the RepeatModeler tool (http://www.repeatmasker.org/RepeatModeler.html) to screen with default settings. From the 353 RepeatModeler-defined elements, only those with >100 full-length hits within scaffolds of the tarsier genome and a length >60 bases were analysed in more detail (for example, for the presence of element flanking target site duplications indicating retroposition).

### Numt screening

We used different strategies to find numts using pairwise and multi-way genome alignments and the GPAC tool^34^ (Supplementary Information).

### Positive gene selection

To detect positive selection, we compared orthologous genes between species (Supplementary Table 8). Protein and CDS sequences were extracted from the Ensembl, UCSC, or NCBI databases. To identify orthologous groups we used orthoMCL^60^ (Supplementary Information). From all detected orthologous groups, we selected all unique groups/genes per species. For these one-to-one orthologous gene groups (*N*=2,099), we extracted the nucleotide sequences for subsequent analysis steps. For each of the 2,099 groups, we built multi-way alignments using GUIDANCE^61^, which, under default parameters, removes sequence positions under a confidence score of 0.93.

Positively selected genes were detected with a branch-site test (H0 and H1) using the codeml package as implemented in PAML^38^. The following tree structure was used: (tbe,(oga,(tsy#1,(cja,(mac,(ggo,(ptr,hsa))))))). The species included in the analysis of positive selection are given in Supplementary Table 10. Tarsier (Tsy#1) is the selected phylogenetic branch of interest. After running both models, we conducted likelihood ratio tests for each model and searched for positively selected genes with an alpha-value <5%. Using the branch-site model (Supplementary Table 7), the set of significant genes was used as input into ToppGene^40^ and WebGestalt^62^. Default parameters were used for the ToppFunn tool within ToppGene and all diseases associated with *P* values <0.05 were output for manual examination. We next assessed enrichment of gene functional clusters under positive natural selection using WebGestalt. Entrez Gene IDs were input as gene symbols, with the organism of interest set to *H. sapiens* using the genome as the reference set. Significant Gene Ontology categories^63^, WikiPathways^64^ and KEGG Pathways^65^ were reported using a hypergeometric test, and the significance level was set at *P*<0.05. For both the ToppGene and the WebGestalt analyses, we implemented the Benjamini and Hochberg multiple test adjustment^39^ to control for false discovery.

### Pairwise sequentially Markovian coalescent

To perform a PSMC analysis^44^, we used a ∼164-GB binary version (BAM) of the SAM file (Sequence Alignment/Map format) a tab-delimited text file with alignment data produced by mapping the fragment reads against their scaffolds and contigs of *T. syrichta*. To analyse the diploid autosomes, we removed the tarsier sex chromosomes after detection in human-tarsier pairwise alignments using annotation from the human genome. For this task, we used a custom python script, which is available by request. In a local version of PSMC we used these parameter settings: a generation interval of seven years (IUCN http://www.iucnredlist.org/details/21492/0), a human mutation rate of 1.1e−08 (ref. 44) and then performed a total of 100 rounds of bootstrapping (see Supplementary Information for specific parameter settings; also see PSMC description https://github.com/lh3/psmc).

### Data availability

The assembled genome of *T. syrichta* is available in the GenBank assembly archive under accession number GCA_000164805.2. All relevant sequence data can be obtained under NCBI BioProject accession number PRJNA20339.

## Additional information

**How to cite this article**: Schmitz, J. *et al*. Genome sequence of the basal haplorrhine primate *Tarsius syrichta* reveals unusual insertions. *Nat. Commun.* **7**, 12997 doi: 10.1038/ncomms12997 (2016).

## Supplementary Material

Supplementary Information Supplementary Figures 1-6, Supplementary Tables 1-10, Supplementary Methods and Supplementary References.

Supplementary Data 1 snoRNA sequence alignments

Supplementary Data 2 miRNA sequence alignments

Supplementary Data 3 Mitochondria sequence alignment

## Figures and Tables

**Table 1:** Repeat element landscape and selected pseudogenes of *Tarsius syrichta* (detailed landscape is presented in Supplementary Table 2).

**Repeat type**	**Families**	**Copies ( × 1000)**	**Genome %**	**Tarsier-specific ( × 1000)**	**Tarsier active families**
LINEs	148	982	19.57	54.4	9
SINEs	16	1697	12.04	175.4	5
TINEs	3	30	0.07	4.7	2
LTR	260	447	5.79	5.9	12
DNA-Trans	219	368	2.63	1.1	8
sRNA pseudo	65	13	0.04	1.7	—
Unclassified	29	1.8	0.01	—	—
Satellite	—	—	1.24	—	—
Low complexity	—	—	2.47	—	—
Total	740	3539	43.85	243.2	36
