# Supplementary material for: Genome sequence of the basal haplorrhine primate Tarsius syrichta reveals unusual insertions
Source: Nat Commun. 2016 Oct 6;7:12997. doi: 10.1038/ncomms12997 (PMC5059674; doi:10.1038/ncomms12997)
Supplement: Supplementary Information — Supplementary Figures 1-6, Supplementary Tables 1-10, Supplementary Methods and Supplementary References. [file ncomms12997-s1.pdf]

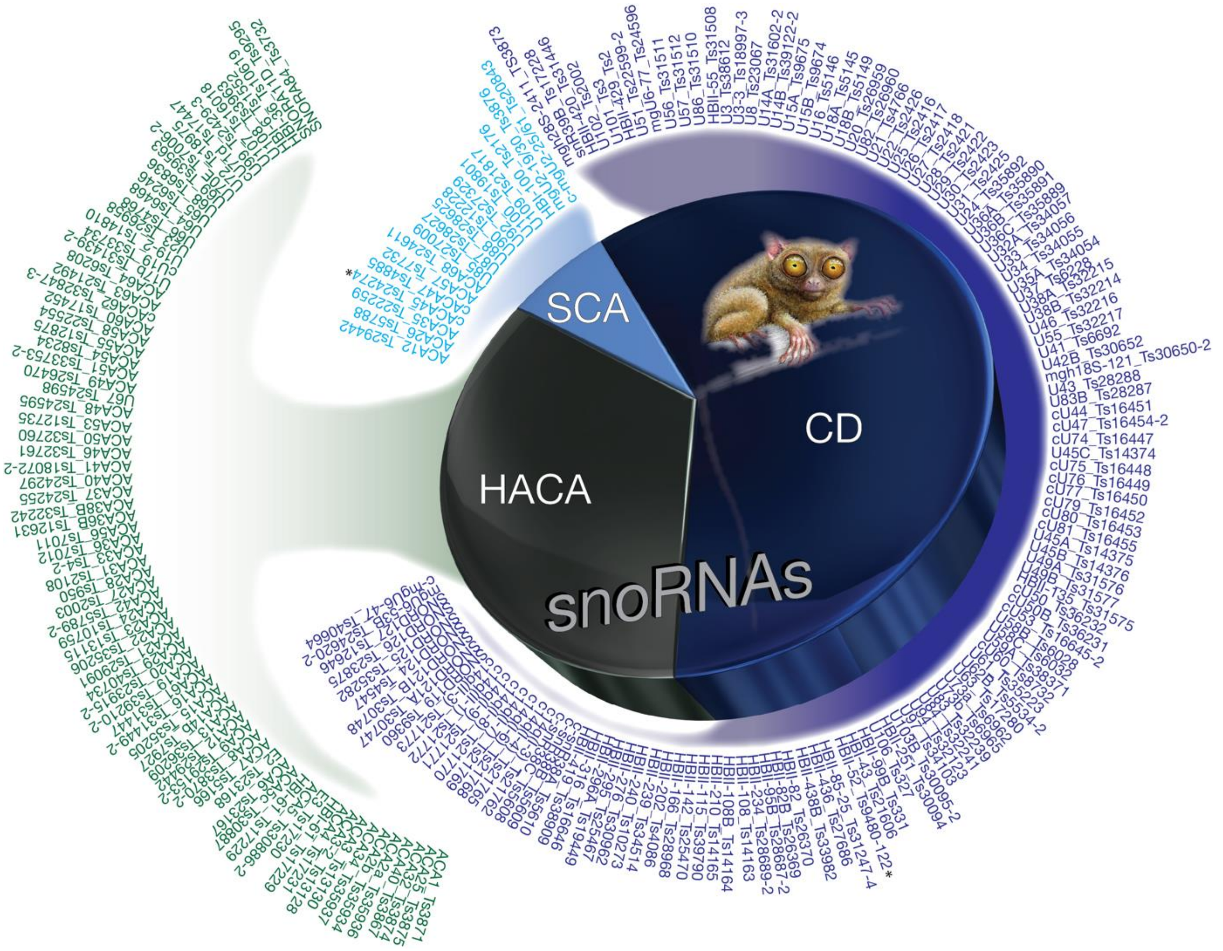

Supplementary Figure 1. The snoRNA transcriptome represents 224 snoRNAs plus 153 isoforms/variants (terminally labeled by additional numbers and separated by a dash, e.g., HBII-52_Ts9480-122* with 122 isoforms/variants). Here, Ts9480 is the *Tarsius syrichta* RNA library candidate name and HBII-52 the human snoRNABase name. The prefix "c" denotes transcription from a snoRNA cluster. Most snoRNAs are encoded intronically and are transcribed together with their host genes. The snoRNAs are divided in scaRNAs (SCA = small Cajal Body localized RNAs containing C/D and H/ACA box motives), CD = C/D box snoRNAs and HACA = H/ACA box snoRNAs. Drawings of animals are provided by Jón Baldur Hlíðberg. Drawing of Tarsiiformes has been reproduced from main text reference [14] with permission.

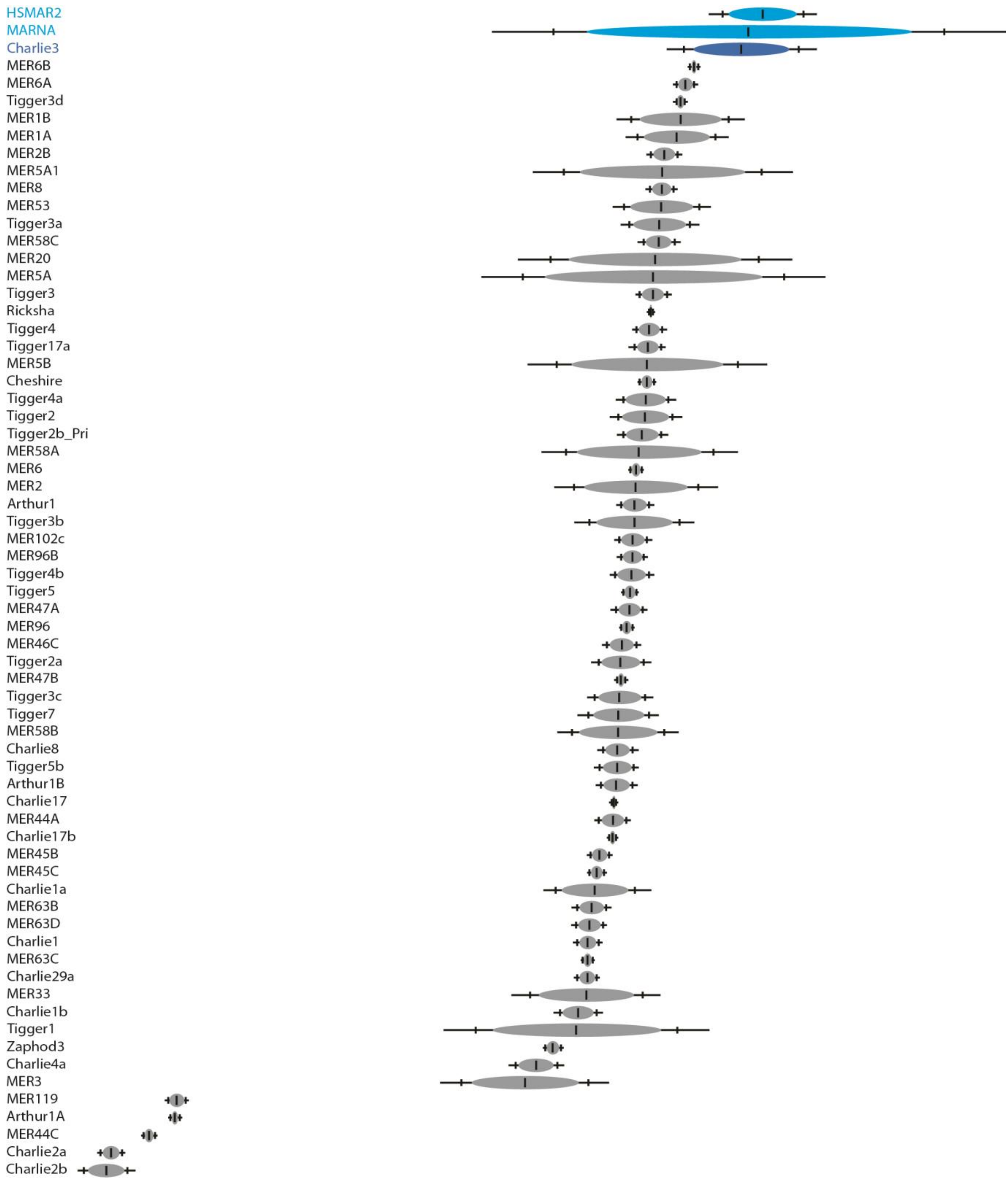

Supplementary Figure 2: Transposition in Transposition (TinT) pattern of DNA transposons derived from the tarsier genome. Potentially active elements are shown in blue. HSMAR2 is a non-autonomous DNA transposon that depends on the transposase of the autonomous MARNA element. The maximum activity period is indicated as a central vertical line in the ovals. The flanking vertical lines represent the 75th percentile activity range, while the end of the lines represent the 99th percentile range of activity. Timescale runs from left to right.

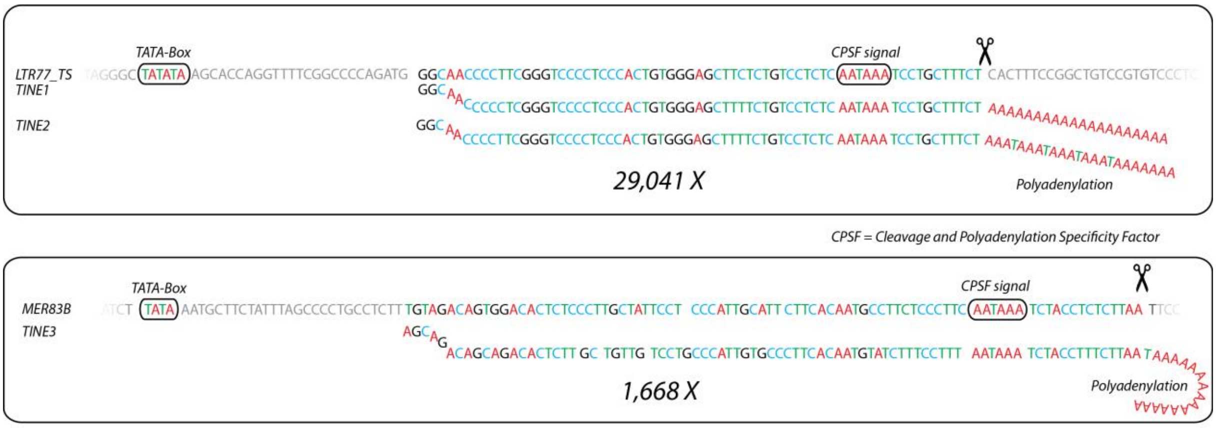

Supplementary Figure 3. The three tarsier-specific, TINE-retroposed, multicopy retropseudogenes (consensus sequences, TINE1, TINE2, TINE3) are represented with their putative source genes, the LTR77_TS and MER82B for TINE1/2 (*top*) and TINE3 (*bottom*), respectively. Note that TINE2 might involve alternative polyadenylation sites within the terminal oligo(A) tail. Hence it might be considered as an alternative cleavage and polyadenylation variant. The LTR/MER gene-specific TATA boxes are used for transcription initiation. The LTR/MER cleavage and polyadenylation specificity factor signals (CPSF) induce cleavage and polyadenylation of the new TINE elements. The tarsier genome contains 29,041 TINE1/2 and 1,668 TINE3 elements.

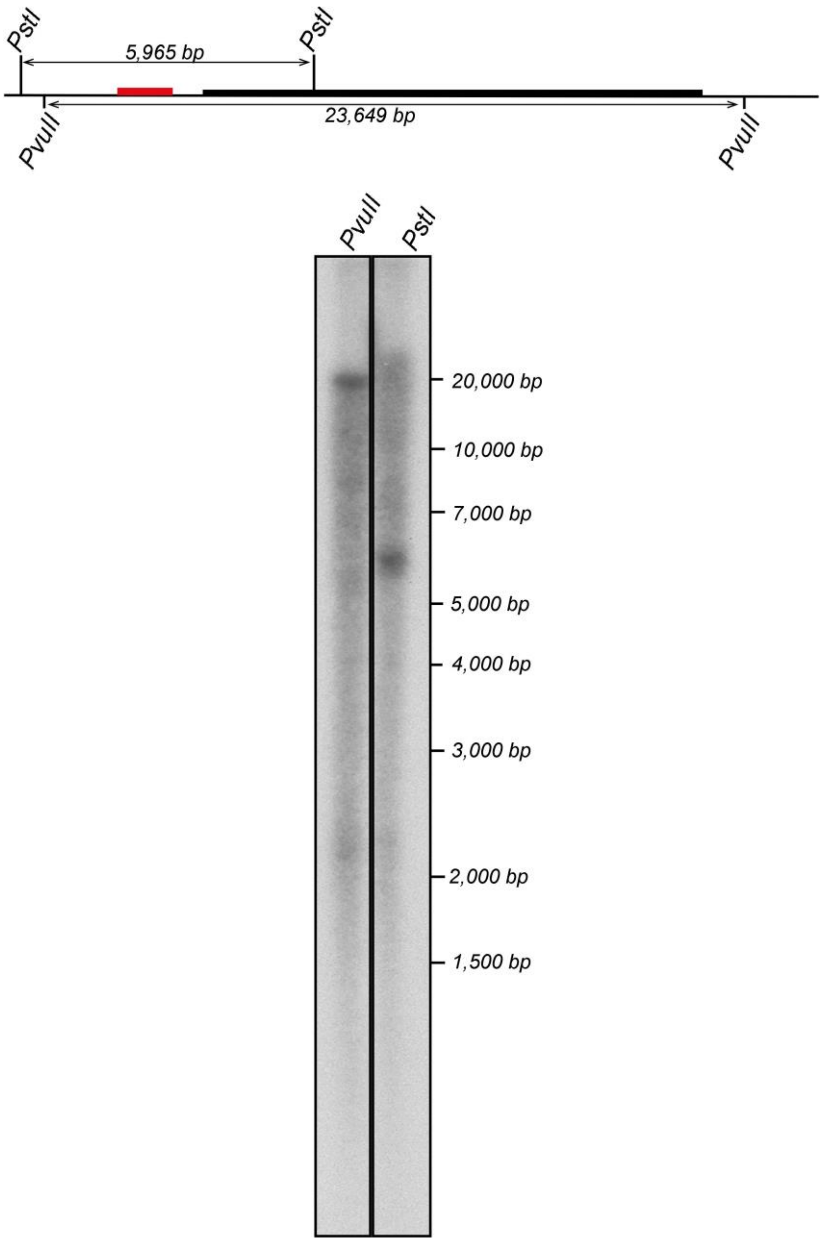

Supplementary Figure 4: Southern blot of the full-length numt sequence region.

PSTI and PVUII digested genomic Tarsius DNA was used to hybridize the red-labeled probe with PCR amplification primers in capital letters (TGAAGGACACAGAGGAATCCAAGATAACCtttggacttggacctaagtatggaagtgagtggtaataccagttactaaggggaaatgcgggaagattagatatgtcaaaatcaggagttctatttggacacattgtgtttgagatgtttattgaaacccaagagatgttgagtaggacgttgggtatttgaatctaaggttcaggggagatatatatttgtgagttgttagcataatgaggtttatggggttatatgagatcactcattaagagttcaggaagagaaagactgaggggtgatcaataagaatgaagggtaataaagtgtattgtccatgaagtcaagtgaaaaaaaagtatttcaagaaggagggaatgagtagctgtttggaatattgctgaaaggcagggtaaaatgaattggtaactgacattggatttggtaaggggtagtgacctttataagtgcagtttctgtgggctgatgagaaggaagatctgaccaaagtaagttagctagggcatggGATGCAATGTGGAGAGTGAGTAATG) to the flanking region of the detected complete mitochondrial insertion. Hybridization signals are shown in the lower part of the figure for both digestion experiments (about 23,000 bps and 6,000 bps) in addition to a size marker.

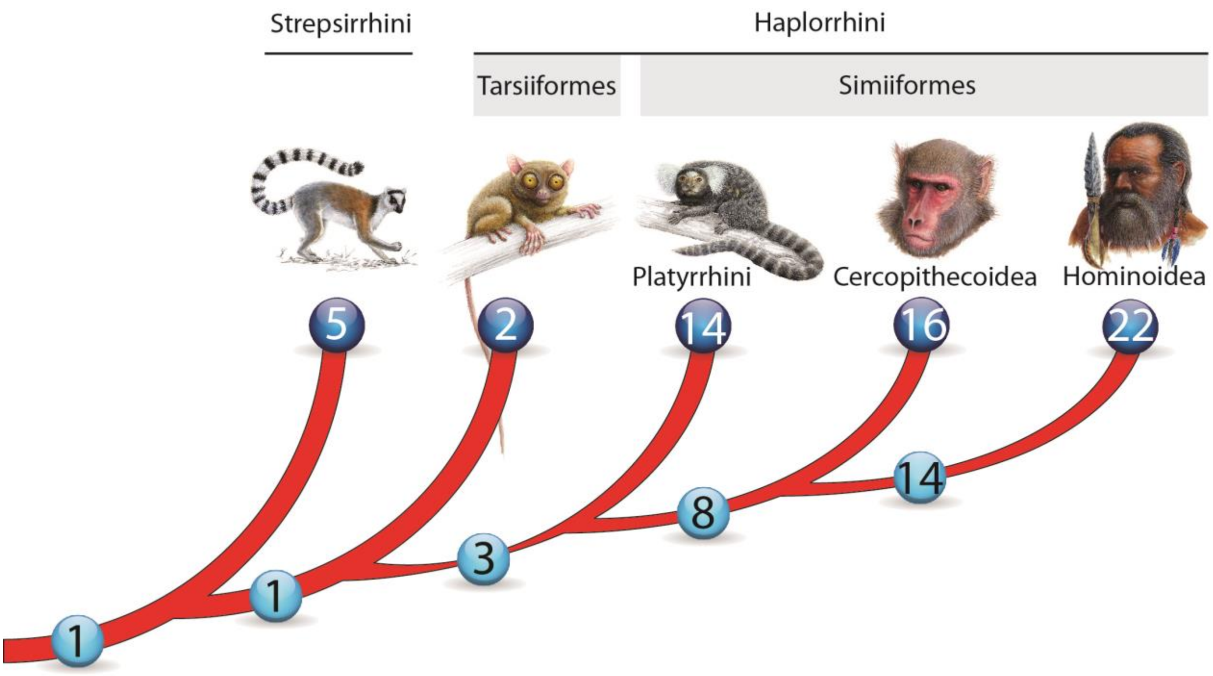

Supplementary Figure 5. Detection of numt insertions in selected primate and internodal lineages. In comparison to the small number of numts that we found in the tarsier (2), we detected the same low number in the genome of the strepsirrhine *Microcebus murinus* representative for other Strepsirrhini and just one numt common for all primates. Fourteen, 16, and 22 lineage specific numts were detected in *Callithrix jacchus*, *Macaca mulatta* and *Homo sapiens*, respectively. Few numts we derived for the ancestral lineages of Haplorrhini (1) and Anthropoidea (3) and a somewhat higher number was reconstructed for the common ancestor of Catarrhini (8) and human plus chimpanzee (14) (see Supplementary Fig. 3). Drawings of animals are provided by Jón Baldur Hlíðberg. Drawings of Tarsiiformes and Hominoidea have been reproduced from main text reference [14] with permission.

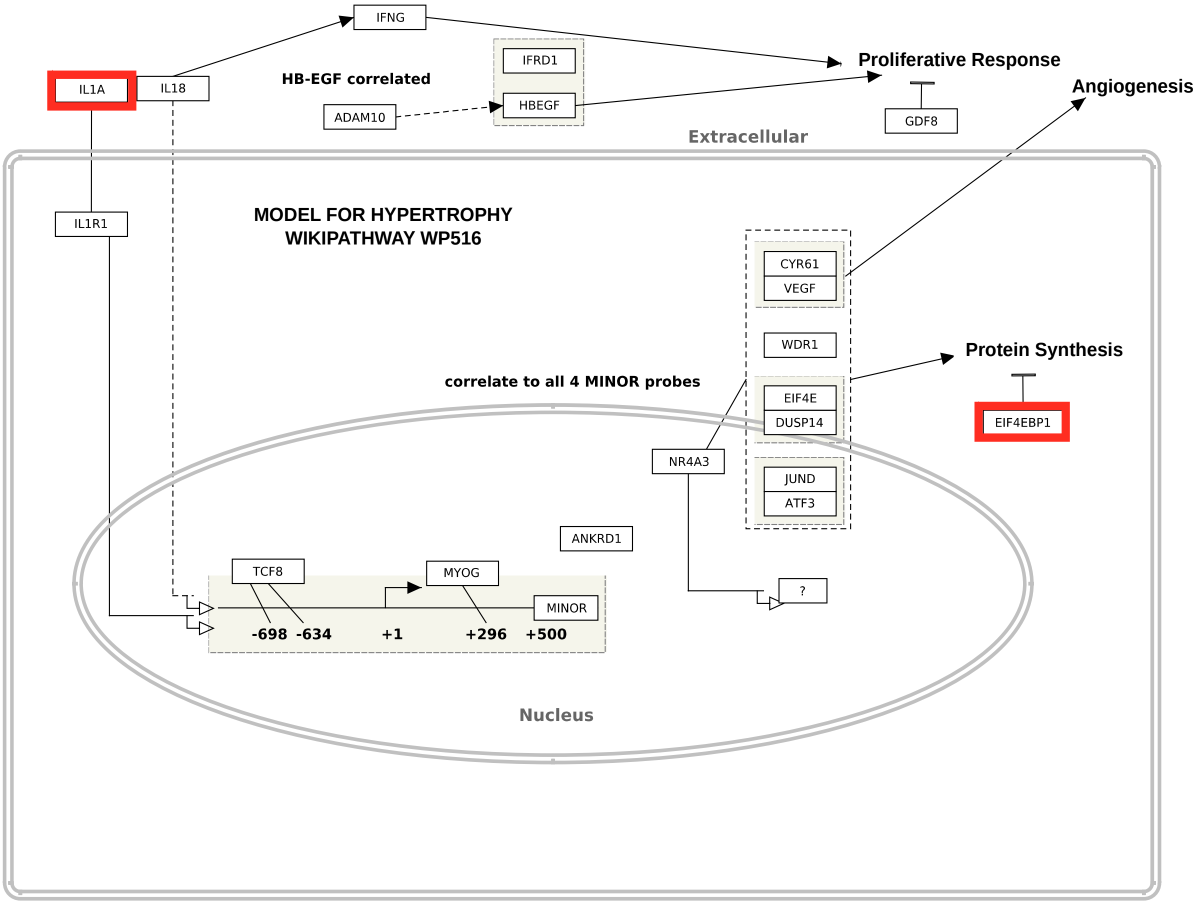

Supplementary Figure 6. A model for hypertrophy (Wikipathway WP516) depicts the role of two positively selected tarsier genes (*IL1A* and *EIF4EBP1*) within the pathway. This pathway is known to regulate skeletal muscle, which typically occurs as tissue growth through increased cell size. *EIF4EBP1* mediates the regulation of protein translation by hormones, growth factors and other stimuli in the cytoplasm, while *IL1A* stimulates cell proliferation by inducing growth factor activity.

Supplementary Table 1. Assembly metrics for other primates using varied sequencing technologies

Common Name	Assembly Version	N50 Contig kb	N50 Scaffold Mb	Assembly Size Gb	Gene count
Chimpanzee	Pan_tro 2.1.4	50	9.0	3.3	18,759
Gorilla	gorGor3.1	12	0.9	3.0	20,962
Orangutan	P_pygmaeus2.0.2	16	0.7	3.4	20,424
Gibbon	Nleu1.0	35	52.0	2.9	18,575
Rhesus macaque	MacaMv7	54	3.0	3.2	21,905
Cynomolgus macaque	MFas 5.0	86	88.0	2.9	NA
Marmoset	C_jacchus3.2.1	29	5.0	2.9	20,993
Bushbaby	OtoGar3.0	27	13.0	2.5	19,506
Baboon	PapAnu2.0	40	0.5	2.9	19,210
Squirrel monkey	saiBol1	38	18.0	2.6	NA
Snub-nose monkey	Rrox V1	77	1.5	2.9	NA
Tarsier	Tarsius_syrichta-2.0.1	38	0.4	3.4	20,820
Bonobo	Pan_pan	66	10.0	2.9	20,826

Supplementary Table 2. Tarsier snoRNAs in comparison to human

Host gene	human	tarsier	draft name	length	reads	class	human					
							target site 1		target site 2		target site 3	
1	2	3	4	5	6	7	8		9		10	
AK128061	ACA1	ACA1_Ts3871	tar_cand_3871_185_83	140	83	HACA	28S	U4441				
	ACA25	ACA25_Ts3875	tar_cand_3875_187_307	132	307	HACA	18S	U801	18S	U814		
	ACA32	ACA32_Ts3874	tar_cand_3874_195_92	120	92	HACA	28S	U1847				
	ACA40	ACA40_Ts3867	tar_cand_3867_134_172	129	172	HACA	28S	U4546	18S	U1174		
	mgh28S-2411	mgh28S-2411_Ts3873	tar_cand_3873_168_6446	72	6446	CD	28S	G2411				
FLJ20436	ACA2a	ACA2a_Ts35936	tar_cand_35936_176_675	135	675	HACA	28S	U4263	28S	U4282		
	ACA2b	ACA2b_Ts35934	tar_cand_35934_191_99	137	99	HACA	28S	U4263	28S	U4282		
	ACA34	ACA34_Ts35937	tar_cand_35937_183_1064	141	1064	HACA	28S	U4269	28S	U4282		
RPL27A	ACA3	ACA3_Ts13130	tar_cand_13130_188_2439	130	2439	HACA	28S	U3899	28S	U3938		
	ACA3-2	ACA3-2_Ts13128	tar_cand_13128_194_4016	131	4016	HACA	28S	U3899				
EIF4A2	ACA4	ACA4_Ts17231	tar_cand_17231_326_2881	140	2881	HACA	18S	U1347				
	HBI-61	HBI-61_Ts17229	tar_cand_17229_356_2538	176	2538	HACA	28S	U4606				
	snR39B	snR39B_Ts17228	tar_cand_17228_339_87325	70	87325	CD	28S	G1509				
	E3	E3_Ts17230	tar_cand_17230_208_9634	208	9634	HACA	28S	U4390				
TBRG4	ACA5	ACA5_Ts30886	tar_cand_30886_136_49	134	49	HACA	18S	U1238	28S	U1625		

		ACA5_Ts30888	tar_cand_30888_277_27494	134	27494	HACA						
	ACA5c	ACA5c_Ts30887	tar_cand_30887_187_2358	136	2358	HACA	18S	U1238	18S	U1625		
RPSA	ACA6	ACA6_Ts23167	tar_cand_23167_205_1134	205	1134	HACA	28S	U3616				
LAMR1	E2	E2_Ts23168	tar_cand_23168_205_1654	155	1654	HACA	28S	U3830	28S	U3832		
**1**	**2**	**3**	**4**	**5**	**6**	**7**	**8**		**9**		**10**	
RPL32	ACA7	ACA7_Ts5166	tar_cand_5166_188_1873	140	1873	HACA	28S	U1569	28S	U1779		
MGC5306	ACA8	ACA8_Ts3870	tar_cand_3870_328_20904	139	20904	HACA	18S	U1056	18S	U1081	28S	U3832
		ACA8_Ts39088	tar_cand_39088_166_724	137	724	HACA						
SNHG15	ACA9	ACA9_Ts29153	tar_cand_29153_151_36	134	36	HACA	28S	U1670	28S	U1769		
		ACA9_Ts30890	tar_cand_30890_171_291	133	291	HACA						
POLA	ACA12	ACA12_Ts29442	tar_cand_29442_188_5247	129	5247	SCARNA23	U6	U40				
TIGA1	ACA13	ACA13_Ts36209	tar_cand_36209_170_1433	133	1433	HACA	18S	U1248				
TIOMM20	ACA14b	ACA14b_Ts7927	tar_cand_7927_231_4764	136	4764	HACA	18S	U966				
CCT6A	ACA15	ACA15_Ts35205	tar_cand_35205_162_190	135	190	HACA	18S	U1367				
AF277181	ACA16	ACA16_Ts31449	tar_cand_31449_244_623	133	623	HACA	28S	U4412				
	ACA44	ACA44_Ts31448	tar_cand_31448_199_20551	131	20551	HACA	18S	U822	18S	U686		

	ACA61	ACA61_Ts31447	tar_cand_31447_196_27106	133	27106	HACA	28S	U2495				
	HBII-420	HBII-420_Ts31446	tar_cand_31446_126_14385	80	14385	CD	28S	A2774				
EIF3S10	ACA19	ACA19_Ts39210	tar_cand_39210_152_45	133	45	HACA	28S	U3618	28S	U3709		
		ACA19_Ts39212	tar_cand_39212_176_708	129	708	HACA						
**1**	**2**	**3**	**4**	**5**	**6**	**7**	**8**		**9**		**10**	
TCP1	ACA20	ACA20_Ts23915	tar_cand_23915_161_101	130	101	HACA	18S	U651				
		ACA20_Ts40735	tar_cand_40735_177_357	130	357	HACA						
	ACA29	ACA29_Ts40734	tar_cand_40734_193_335	137	335	HACA	?					
RPL23	ACA21	ACA21_Ts29091	tar_cand_29091_249_2851	136	2851	HACA	28S	U4401	28S	U4470		
BC052238	ACA22	ACA22_Ts35206	tar_cand_35206_180_331	134	331	HACA	28S	U4966	28S	U4975		
IPO7	ACA23	ACA23_Ts13115	tar_cand_13115_256_676	188	676	HACA	28S	U3737	28S	U4331		
SNHG8	ACA24	ACA24_Ts10759	tar_cand_10759_173_816	131	816	HACA	18S	U863	18S	U609		
KIAA0907	ACA26	ACA26_Ts5788	tar_cand_5788_189_1511	129	1511	SCARNA4	U2	U41	U2	U39		
	ACA42	ACA42_Ts5789	tar_cand_5789_204_5145	135	5145	HACA	18S	U572	18S	U109		
		ACA42_Ts33368	tar_cand_33368_181_3243	134	3243	HACA						
RPL21	ACA27	ACA27_Ts2003	tar_cand_2003_186_23363	126	23363	HACA	28S	U3694	28S	U4522		
	U102	U102_Ts2002	tar_cand_2002_122_142218	122	142218	CD	28S	G4020				

EIF5	ACA28	ACA28_Ts950	tar_cand_950_217_1457	126	1457	HACA	18S	U815	18S	U866		
TPT1	ACA31	ACA31_Ts2108	tar_cand_2108_143_75	126	75	HACA	18S	U218	28S	U3713		
RPS12	ACA33	ACA33_Ts4	tar_cand_4_166_511	131	511	HACA	28S	U4966				
		ACA33_Ts21993	tar_cand_21993_127_43	127	43	HACA						
	U101	U101_Ts3	tar_cand_3_130_71560	74	71560	CD	?					
	HBII-429	HBII-429_Ts2	tar_cand_2_138_1830743	76	1830743	CD	18S	G436				
**1**	**2**	**3**	**4**	**5**	**6**	**7**	**8**		**9**		**10**	
PPP1R8	ACA35	ACA35_Ts22259	tar_cand_22259_217_3060	165	3060	SCARNA1	U2	U89				
DKC1	ACA36	ACA36_Ts7012	tar_cand_7012_183_1162	133	1162	HACA	18S	U1244	18S	U105		
	ACA56	ACA56_Ts7011	tar_cand_7011_148_32	129	32	HACA	28S	U1664				
RAB3-GAP150	ACA36B	ACA36B_Ts12631	tar_cand_12631_177_595	131	595	HACA	18S	U105	18S	U1244		
NOL11	ACA38B	ACA38B_Ts32242	tar_cand_32242_198_41	131	41	HACA	?					
MBD2	ACA37	ACA37_Ts24255	tar_cand_24255_185_13425	132	13425	HACA	28S	U4643				
AK128061	ACA40	ACA40_Ts24297	tar_cand_24297_88_31	87	31	HACA	18S	U1174	28S	U4546		
EEF1B2	ACA41	ACA41_Ts18072	tar_cand_18072_171_554	134	554	HACA	18S	U1643				
		ACA41_Ts22598	tar_cand_22598_212_1110	137	1110	HACA						

	U51	U51_Ts22599	tar_cand_22599_174_4186	70	4186	CD	28S	A1511				
		U51_Ts7457	tar_cand_7457_69_28	68	28	CD						
EST Cluster	ACA45	ACA45_Ts24274	tar_cand_24274_211_565	126	565	SCARNA15	U2	U37				
CNOT1	ACA46	ACA46_Ts32761	tar_cand_32761_166_2535	137	2535	HACA	18S	U649				
	ACA50	ACA50_Ts32760	tar_cand_32760_311_793	136	793	HACA	18S	U34	18S	U105		
EST cluster	ACA47	ACA47_Ts4885	tar_cand_4885_237_1955	186	1955	SCARNA16	U1	U5				
SLC25A3	ACA53	ACA53_Ts12735	tar_cand_12735_294_3759	247	3759	HACA	?					
**1**	**2**	**3**	**4**	**5**	**6**	**7**	**8**		**9**		**10**	
EIF4A1	ACA48	ACA48_Ts24595	tar_cand_24595_390_274	135	274	HACA	28S	U3797				
	U67	U67_Ts24598	tar_cand_24598_180_417	142	417	HACA	18S	U1445				
	mgU6-77	mgU6-77_Ts24596	tar_cand_24596_204_26012	151	26012	CD	U6	C77	28S	C3787		
EP400	ACA49	ACA49_Ts26470	tar_cand_26470_199_312	134	312	HACA	?					
NOL5A	ACA51	ACA51_Ts33753	tar_cand_33753_173_758	132	758	HACA	?					
		ACA51_Ts31509	tar_cand_31509_170_598	129	598	HACA						
	U56	U56_Ts31511	tar_cand_31511_187_9004	67	9004	CD	18S	C517				
	U57	U57_Ts31512	tar_cand_31512_116_51356	70	51356	CD	18S	A99				
	U86	U86_Ts31510	tar_cand_31510_96_64	85	64	CD	?					
	HBII-55	HBII-55_Ts31508	tar_cand_31508_116_8204	71	8204	CD	18S	U1288				

NAP1L4	ACA54	ACA54_Ts8234	tar_cand_8234_183_13919	122	13919	HACA	28S	U3801	28S	U4539		
PABPC4	ACA55	ACA55_Ts12875	tar_cand_12875_187_12014	135	12014	HACA	18S	U36				
CHD4	ACA57	ACA57_Ts7732	tar_cand_7732_186_825	139	825	SCARNA11	U5	U43				
MRPL3	ACA58	ACA58_Ts22554	tar_cand_22554_180_554	137	554	HACA	28S	U3823				
LOC128439	ACA60	ACA60_Ts17452	tar_cand_17452_224_44072	134	44072	HACA	18S	U1004				
EST cluster	ACA62	ACA62_Ts32847	tar_cand_32847_172_13070	126	13070	HACA	18S	U34	18S	U105		
		ACA62_Ts33863	tar_cand_33863_176_3313	126	3313	HACA						
		ACA62_Ts33864	tar_cand_33864_183_3742	126	3742	HACA						
**1**	**2**	**3**	**4**	**5**	**6**	**7**	**8**		**9**		**10**	
ATP2B4	ACA63	ACA63_Ta21492	tar_cand_21492_166_1130	123	1130	HACA	18S	U814				
DKFZp686N06141	ACA64	ACA64_Ts6208	tar_cand_6208_167_1848	124	1848	HACA	28S	U4331				
CHD3	ACA68	ACA68_Ts24611	tar_cand_24611_279_51	136	51	SCARNA21	U12	U19				
trans unit	U3	U3_Ts38612	tar_cand_38612_248_30738	215	30738	CD	?					

	U3-3	U3-3_Ts18997	tar_cand_18997_230_15551	217	15551	CD	?					
		U3-3_Ts18317	tar_cand_18317_88_33	88	33	CD	?					
	U8	U8_Ts23067	tar_cand_23067_158_3430	138	3430	CD	?					
RPS13	U14A	U14A_Ts31602	tar_cand_31602_122_358	91	358	CD	18S	C462				
		U14A_Ts39123	tar_cand_39123_228_15024	88	15024	CD						
	U14B	U14B_Ts39122	tar_cand_39122_122_1034	87	1034	CD	18S	C462				
		U14B_Ts31600	tar_cand_31600_213_926	92	926	CD						
RPS3	U15A	U15A_Ts9675	tar_cand_9675_217_1947	147	1947	CD	28S	A3764				
	U15B	U15B_Ts9674	tar_cand_9674_236_6783	146	6783	CD	28S	A3764				
RPL4	U16	U16_Ts5146	tar_cand_5146_158_90639	101	90639	CD	18S	A484				
	U18A	U18A_Ts5145	tar_cand_5145_182_278946	70	278946	CD	28S	A1313				
	U18B	U18B_TS5149	tar_cand_5149_164_1129	70	1129	CD	28S	A1313				
**1**	**2**	**3**	**4**	**5**	**6**	**7**	**8**		**9**		**10**	
U17HG	U17b	U17b_Ts31439	tar_cand_31439_312_2681	205	2681	HACA	?					
		U17b_Ts31438	tar_cand_31438_275_7258	204	7258	HACA	?					
U19H noncoding	U19	U19_Ts33734	tar_cand_33734_263_665	203	665	HACA	28S	U3741	28S	U3743	U3	U8
ATP6VOE	U19-2	U19-2_Ts14810	tar_cand_14810_259_760	207	760	HACA	28S	U3741	28S	U3743		

NCL	U20	U20_Ts26959	tar_cand_26959_121_20893	79	20893	CD	18S	U1804				
	U23	U23_Ts26958	tar_cand_26958_287_342	137	342	HACA	18S	U93				
	U82/Z25	U82_Ts26960	tar_cand_26960_119_58359	75	58359	CD	18S	A1678				
RPL5	U21	U21_Ts4766	tar_cand_4766_149_6021	95	6021	CD	28S	G1303				
	U66	U66_Ts4768	tar_cand_4768_175_304	133	304	HACA	18S	U119				
UHG	U22	U22_Ts2426	tar_cand_2426_193_143998	125	143998	CD	?					
	U25	U25_Ts2416	tar_cand_2416_109_4637	68	4637	CD	18S	G1490				
	U26	U26_Ts2417	tar_cand_2417_114_33281	75	33281	CD	28S	A389				
	U27	U27_Ts2418	tar_cand_2418_196_556356	71	556356	CD	18S	A27				
	U28	U28_Ts2421	tar_cand_2421_104_1282	75	1282	CD	18S	C1391				
	U29	U29_Ts2422	tar_cand_2422_93_454	65	454	CD	28S	A4493				
	U30	U30_Ts2423	tar_cand_2423_187_895319	71	895319	CD	28S	A3804				
	U31	U31_Ts2425	tar_cand_2425_175_91210	68	91210	CD	28S	A4166				
RPL7A	U24	U24_Ts35892	tar_cand_35892_204_74125	76	74125	CD	28S	C2338	28S	C2352		
	U36A	U36A_Ts35890	tar_cand_35890_157_50	72	50	CD	18S	A668				
	U36B	U36B_Ts35891	tar_cand_35891_167_47861	69	47861	CD	18S	A668				
	U36C	U36C_Ts35889	tar_cand_35889_124_21412	67	21412	CD	28S	A3703				
**1**	**2**	**3**	**4**	**5**	**6**	**7**	**8**		**9**		**10**	
RPL13A	U32A	U32A_Ts34057	tar_cand_34057_269_168303	77	168303	CD	18S	G1328	28S	A1511		
	U33	U33_Ts34056	tar_cand_34056_224_13088	83	13088	CD	18S	U1326				

	U34	U34_Ts34055	tar_cand_34055_122_132148	66	132148	CD	28S	U2824				
	U35A	U35A_Ts34054	tar_cand_34054_142_11246	85	11246	CD	28S	C4506				
EEF2	U37	U37_Ts6228	tar_cand_6228_154_98942	65	98942	CD	28S	A3697				
RPS8	U38A	U38A_Ts32215	tar_cand_32215_121_2886	71	2886	CD	28S	A1858				
	U38B	U38B_Ts32214	tar_cand_32214_222_11122	69	11122	CD	28S	A1858				
	U46	U40_Ts32216	tar_cand_32216_162_285335	98	285335	CD	28S	A3739				
	U55	U55_Ts32217	tar_cand_32217_115_88133	73	88133	CD	28S	C2791				
TNPO2	U41	U41_Ts6692	tar_cand_6692_124_55147	70	55147	CD	28S	U4276				
RPL23A	U42B	U42B_Ts30652	tar_cand_30652_122_177079	66	177079	CD	18S	U116				
	mgh18S-121	mgh18S-121_Ts30650	tar_cand_30650_179_60013	69	60013	CD	18S	U	121			
		mgh18S-121_Ts37991	tar_cand_37991_108_715	68	715	CD						
RPL3	U43	U43_Ts28288	tar_cand_28288_136_12632	63	12632	CD	18S	C1703				
	U83B	U83B_Ts28287	tar_cand_28287_143_5231	92	5231	CD	?					
**1**	**2**	**3**	**4**	**5**	**6**	**7**	**8**		**9**		**10**	
AL110141	U44	U44_Ts16451	tar_cand_16451_109_250383	63	250383	CD	18S	A166				
	U47	U47_Ts16454	tar_cand_16454_175_46841	66	46841	CD	28S	C3866				
		U47_Ts18172	tar_cand_18172_74_21	66	21	CD						

	U74	U74_Ts16447	tar_cand_16447_110_4278	66	4278	CD	28S	C3820				
	U75	U75_Ts16448	tar_cand_16448_91_1871	60	1871	CD	28S	C4032				
	U76	U76_Ts16449	tar_cand_16449_221_6532	80	6532	CD	28S	A2350				
	U77	U77_Ts16450	tar_cand_16450_85_2242	70	2242	CD	28S	A1521				
	U79	U79_Ts16452	tar_cand_16452_130_30413	83	30413	CD	28S	A3809				
	U80	U80_Ts16453	tar_cand_16453_161_839361	78	839361	CD	28S	A1521	28S	G1612		
	U81	U81_Ts16455	tar_cand_16455_133_49130	77	49130	CD	28S	A391				
RABGGTB	U45A	U45A_Ts14375	tar_cand_14375_132_41502	83	41502	CD	18S	A159	18S	U172		
	U45B	U45B_Ts14376	tar_cand_14376_104_10859	71	10859	CD	18S	U172				
	U45C	U45C_Ts14374	tar_cand_14374_123_7618	78	7618	CD	18S	A159				
DKFZ p686I23148	U49A	U49A_Ts31576	tar_cand_31576_122_32990	71	32990	CD	28S	C4426				
	U49B	U49B_Ts31577	tar_cand_31577_190_13592	48	13592	CD	28S	C4426				
	HBII-135	HBII-135_Ts31575	tar_cand_31575_126_15265	73	15265	CD	18S	U627				
EST cluster	U50	U50_Ts36232	tar_cand_36232_279_84356	72	84356	CD	28S	C2848	28S	G2863		
	U50B	U50B_Ts36231	tar_cand_36231_185_4261	72	4261	CD	?					
WD-repeat protien 43	U53	U53_Ts16645	tar_cand_16645_115_99568	78	99568	CD	28S	C3848				
		U53_Ts16644	tar_cand_16644_114_851	78	851	CD						
RPL17	U58B	U58B_Ts6028	tar_cand_6028_111_1062	65	1062	CD	28S	G4198				

	U58C	U58C_Ts6032	tar_cand_6032_110_56372	65	56372	CD	28S	U4197	28S	G4198		
**1**	**2**	**3**	**4**	**5**	**6**	**7**	**8**		**9**		**10**	
ATP5B	U59A	U59A_Ts38371	tar_cand_38371_233_2892	75	2892	CD	18S	A1031				
EST cluster	U60	U60_Ts8732	tar_cand_8732_212_3251	83	3251	CD	28S	G4340				
RBMX	U61	U61_Ts35223	tar_cand_35223_144_69199	72	69199	CD	18S	U1442				
AB011087	U62B	U62B_Ts5534	tar_cand_5534_140_561	86	561	CD	18S	A590				
		U62B_Ts5535	tar_cand_5535_114_679	86	679	CD						
HSPA9B	U63	U63_Ts17280	tar_cand_17280_113_29478	70	29478	CD	28S	A4541				
RPS2	U64	U64_Ts6209	tar_cand_6209_181_6468	133	6468	HACA	28S	U4975				
RPL12	U65	U65_Ts6246	tar_cand_6246_194_35514	137	35514	HACA	28S	U4373	28S	U4427		
RPL18A	U68	U68_Ts6836	tar_cand_6836_232_56181	131	56181	HACA	28S	U4393				
RPL39	U69	U69_Ts39563	tar_cand_39563_170_182	133	182	HACA	18S	U36	5.8S	U69		
RAB30	U70E	U70E_Ts7006	tar_cand_7006_229_2204	133	2204	HACA	18S	U1692				
		U70E_Ts7740	tar_cand_7740_100_167	96	167	HACA						
	U72	U72_Ts18975	tar_cand_18975_197_1051	131	1051	HACA	5.8S	U55				

spliced EST	U71d	U71d_Ts17449	tar_cand_17449_181_457	138	457	HACA	18S	U406				
RPS3a	U73a	U73a_Ts6962	tar_cand_6962_119_89395	69	89395	CD	?					
	U73b	U73b_Ts6965	tar_cand_6965_115_381809	80	381809	CD	?					
**1**	**2**	**3**	**4**	**5**	**6**	**7**	**8**		**9**		**10**	
BAT1	U83	U83_Ts32239	tar_cand_32239_144_22320	77	22320	CD	?					
	U84	U84_Ts32241	tar_cand_32241_134_918	78	918	CD	?					
CNAP1	U85	U85_Ts27009	tar_cand_27009_358_513	328	513	SCARNA10	U5	U46	U5	C45		
APG16L	U87	U87_Ts28627	tar_cand_28627_286_587	277	587	SCARNA5	U5	U41	U4	A65		
	U88	U88_Ts28625	tar_cand_28625_278_916	269	916	SCARNA6	U5	U41				
KPNA4	U90	U90_Ts12228	tar_cand_12228_343_379	323	379	SCARNA7	U1	A70				
FAM29A	U92	U92_Ts27329	tar_cand_27329_176_728	131	728	SCARNA8	U2	U34	U2	U44		
FLJ20758	U94	U94_Ts32123	tar_cand_32123_302_24427	138	24427	CD	U6	C62				
EIF4G2	U97	U97_Ts34103	tar_cand_34103_191_782	137	782	CD	?					
LOC751071	U99	U99_Ts2429	tar_cand_2429_218_13097	148	13097	HACA	18S	U1004				
		U99_Ts18920	tar_cand_18920_214_27	149	27	HACA						

		U99_Ts37130	tar_cand_37130_154_316	148	316	HACA						
FLJ20516	U100	U100_Ts19801	tar_cand_19801_214_1141	137	1141	SCARNA14	U2	U7				
PUM1	U103B	U103B_Ts30095	tar_cand_30095_155_1835	88	1835	CD	18S	G601				
		U103B_Ts30097	tar_cand_30097_117_1157	87	1157	CD						
	HBII-251	HBII-251_Ts30094	tar_cand_30094_110_1106	75	1106	CD	18S	G601				
C20orf199	U106	U106_Ts327	tar_cand_327_140_27081	82	27081	CD	?					
	HBII-99B	HBII-99B_Ts331	tar_cand_331_208_279928	91	279928	CD	28S	G3878				
**1**	**2**	**3**	**4**	**5**	**6**	**7**	**8**		**9**		**10**	
MAGED2	U107	U107_Ts16018	tar_cand_16018_99_59	80	59	HACA	?					
CWF19L1	U108	U108_Ts3963	tar_cand_3963_298_460	139	460	HACA	?					
MGC23909	U109	U109_Ts21817	tar_cand_21817_181_257	136	257	SCARNA18	U1	U6				
D28589	HBI-6	HBI-6_Ts1552	tar_cand_1552_157_226	122	226	HACA	28S	U4522				
HTR2C	HBI-36	HBI-36_Ts10619	tar_cand_10619_185_2749	128	2749	HACA	?					
SNX5	HBI-43	HBI-43_Ts21606	tar_cand_21606_284_6492	235	6492	CD	28S	U3797				
RFWD2	HBI-100	HBI-100_Ts2176	tar_cand_2176_198_7511	141	7511	SCARNA3	U6	U40				

SNURF-SNRNP-UBE3A antisense	HBII-52	HBII-52_Ts9480	tar_cand_9480_331_319500	82	319500	CD	serotonin					
		HBII-52_Ts23742	tar_cand_23742_133_160433	82	160433	CD						
		HBII-52_Ts23798	tar_cand_23798_133_877	82	877	CD						
		HBII-52_Ts23783	tar_cand_23783_106_4687	82	4687	CD						
		HBII-52_Ts23772	tar_cand_23772_81_220	82	220	CD						
		HBII-52_Ts23767	tar_cand_23767_138_372	81	372	CD						
		HBII-52_Ts23752	tar_cand_23752_81_125	81	125	CD						
		HBII-52_Ts23708	tar_cand_23708_134_2081	82	2081	CD						
		HBII-52_Ts23740	tar_cand_23740_124_22202	82	22202	CD						
		HBII-52_Ts23696	tar_cand_23696_111_502	82	502	CD						
		HBII-52_Ts23700	tar_cand_23700_137_28863	82	28863	CD						
**1**	**2**	**3**	**4**	**5**	**6**	**7**	**8**		**9**		**10**	
		HBII-52_Ts23686	tar_cand_23686_102_4597	82	4597	CD						
		HBII-52_Ts23690	tar_cand_23690_97_618	81	618	CD						
		HBII-52_Ts23788	tar_cand_23788_160_1024	72	1024	CD						
		HBII-52_Ts23793	tar_cand_23793_81_62	81	62	CD						
		HBII-52_Ts23685	tar_cand_23685_82_67	81	67	CD						
		HBII-52_Ts23765	tar_cand_23765_191_133	82	133	CD						
		HBII-52_Ts23823	tar_cand_23823_104_43	82	43	CD						
		HBII-52_Ts23709	tar_cand_23709_133_43	144	43	CD						
		HBII-52_Ts23703	tar_cand_23703_115_1125	76	1125	CD						
		HBII-52_Ts23717	tar_cand_23717_125_2229	82	2229	CD						
		HBII-52_Ts23730	tar_cand_23730_102_300	82	300	CD						

		HBII-52_Ts23731	tar_cand_23731_122_6441	81	6441	CD			
		HBII-52_Ts23736	tar_cand_23736_176_9338	82	9338	CD			
		HBII-52_Ts23739	tar_cand_23739_81_616	81	616	CD			
		HBII-52_Ts23744	tar_cand_23744_105_1178	82	1178	CD			
		HBII-52_Ts23750	tar_cand_23750_134_8403	82	8403	CD			
		HBII-52_Ts23711	tar_cand_23711_100_202	82	202	CD			
		HBII-52_Ts23778	tar_cand_23778_266_231	82	231	CD			
		HBII-52_Ts23784	tar_cand_23784_166_4967	82	4967	CD			
		HBII-52_Ts23785	tar_cand_23785_83_22	82	22	CD			
		HBII-52_Ts23844	tar_cand_23844_101_907	81	907	CD			
		HBII-52_Ts23754	tar_cand_23754_385_552	82	552	CD			
		HBII-52_Ts23751	tar_cand_23751_109_1793	81	1793	CD			
		HBII-52_Ts23826	tar_cand_23826_130_10914	81	10914	CD			
		HBII-52_Ts23828	tar_cand_23828_137_175	79	175	CD			
		HBII-52_Ts23829	tar_cand_23829_79_24	80	24	CD			
		HBII-52_Ts31048	tar_cand_31048_230_671	72	671	CD			
**1**	**2**	**3**	**4**	**5**	**6**	**7**	**8**	**9**	**10**
		HBII-52_Ts31050	tar_cand_31050_210_5496	74	5496	CD			
		HBII-52_Ts31063	tar_cand_31063_73_140	73	140	CD			
		HBII-52_Ts31075	tar_cand_31075_110_1618	72	1618	CD			
		HBII-52_Ts31083	tar_cand_31083_70_93	70	93	CD			
		HBII-52_Ts31158	tar_cand_31158_160_235	79	235	CD			
		HBII-52_Ts31089	tar_cand_31089_92_20	79	20	CD			
		HBII-52_Ts31092	tar_cand_31092_152_11879	81	11879	CD			

		HBII-52_Ts31116	tar_cand_31116_82_55	80	55	CD			
		HBII-52_Ts31127	tar_cand_31127_182_33	80	33	CD			
		HBII-52_Ts31118	tar_cand_31118_220_378	81	378	CD			
		HBII-52_Ts31135	tar_cand_31135_120_103	79	103	CD			
		HBII-52_Ts31155	tar_cand_31155_102_363	81	363	CD			
		HBII-52_Ts31165	tar_cand_31165_94_1049	81	1049	CD			
		HBII-52_Ts31149	tar_cand_31149_97_88	81	88	CD			
		HBII-52_Ts23832	tar_cand_23832_146_3123	81	3123	CD			
		HBII-52_Ts23834	tar_cand_23834_86_169	82	169	CD			
		HBII-52_Ts23841	tar_cand_23841_205_8109	81	8109	CD			
		HBII-52_Ts31154	tar_cand_31154_112_240	82	240	CD			
		HBII-52_Ts31157	tar_cand_31157_126_117	80	117	CD			
		HBII-52_Ts31246	tar_cand_31246_209_113782	79	113782	CD			
		HBII-52_Ts31109	tar_cand_31109_263_5116	82	5116	CD			
		HBII-52_Ts31088	tar_cand_31088_81_148	81	148	CD			
		HBII-52_Ts32573	tar_cand_32573_272_7560	82	7560	CD			
		HBII-52_Ts32575	tar_cand_32575_230_60190	82	60190	CD			
		HBII-52_Ts32586	tar_cand_32586_136_19564	82	19564	CD			
		HBII-52_Ts32589	tar_cand_32589_102_683	82	683	CD			
		HBII-52_Ts32591	tar_cand_32591_368_10075	82	10075	CD			
		HBII-52_Ts32599	tar_cand_32599_135_12723	82	12723	CD			
**1**	**2**	**3**	**4**	**5**	**6**	**7**	**8**	**9**	**10**
		HBII-52_Ts32601	tar_cand_32601_120_2826	81	2826	CD			
		HBII-52_Ts32604	tar_cand_32604_104_1752	82	17524	CD			

			4									
		HBII-52_Ts32607	tar_cand_32607_80_53	80	53	CD						
		HBII-52_Ts32621	tar_cand_32621_82_122	82	122	CD						
		HBII-52_Ts32626	tar_cand_32626_92_2208	81	2208	CD						
		HBII-52_Ts32629	tar_cand_32629_144_46049	82	46049	CD						
		HBII-52_Ts32630	tar_cand_32630_117_1009	82	1009	CD						
		HBII-52_Ts32631	tar_cand_32631_136_14191	82	14191	CD						
		HBII-52_Ts32633	tar_cand_32633_131_8472	82	8472	CD						
		HBII-52_Ts32634	tar_cand_32634_140_565	82	565	CD						
		HBII-52_Ts32635	tar_cand_32635_121_316	82	316	CD						
		HBII-52_Ts32637	tar_cand_32637_107_60	81	60	CD						
		HBII-52_Ts32639	tar_cand_32639_96_387	82	387	CD						
		HBII-52_Ts32640	tar_cand_32640_87_69	81	69	CD						
		HBII-52_Ts32649	tar_cand_32649_82_381	80	381	CD						
		HBII-52_Ts32652	tar_cand_32652_202_11279	82	11279	CD						
		HBII-52_Ts32657	tar_cand_32657_154_1474	82	1474	CD						
		HBII-52_Ts32660	tar_cand_32660_138_4915	82	4915	CD						
		HBII-52_Ts32666	tar_cand_32666_93_670	82	670	CD						
		HBII-52_Ts32667	tar_cand_32667_167_302	80	302	CD						
		HBII-52_Ts32684	tar_cand_32684_79_194	78	194	CD						
		HBII-52_Ts32685	tar_cand_32685_117_2238	81	2238	CD						
		HBII-52_Ts32691	tar_cand_32691_81_26	81	26	CD						
		HBII-52_Ts32694	tar_cand_32694_188_87	81	87	CD						
		HBII-52_Ts32698	tar_cand_32698_164_580	82	580	CD						

		HBII-52_Ts32704	tar_cand_32704_278_210	82	210	CD			
		HBII-52_Ts32712	tar_cand_32712_126_2435	82	2435	CD			
		HBII-52_Ts32721	tar_cand_32721_106_1074	82	1074	CD			
**1**	**2**	**3**	**4**	**5**	**6**	**7**	**8**	**9**	**10**
		HBII-52_Ts23723	tar_cand_23723_243_343	82	343	CD			
		HBII-52_Ts32580	tar_cand_32580_186_3851	81	3851	CD			
		HBII-52_Ts32605	tar_cand_32605_106_2630	82	2630	CD			
		HBII-52_Ts32682	tar_cand_32682_144_2239	81	2239	CD			
		HBII-52_Ts23802	tar_cand_23802_82_175	82	175	CD			
		HBII-52_Ts31159	tar_cand_31159_79_273	79	273	CD			
		HBII-52_Ts23825	tar_cand_23825_224_17369	81	17369	CD			
		HBII-52_Ts32615	tar_cand_32615_83_227	81	227	CD			
		HBII-52_Ts23697	tar_cand_23697_83_2637	79	2637	CD			
		HBII-52_Ts23682	tar_cand_23682_80_64	80	64	CD			
		HBII-52_Ts32596	tar_cand_32596_80_51	80	51	CD			
		HBII-52_Ts23812	tar_cand_23812_83_4565	62	4565	CD			
		HBII-52_Ts31181	tar_cand_31181_72_200	72	200	CD			
		HBII-52_Ts23843	tar_cand_23843_71_143	71	143	CD			
		HBII-52_Ts31152	tar_cand_31152_65_36	64	36	CD			
		HBII-52_Ts23845	tar_cand_23845_62_171	62	171	CD			
		HBII-52_Ts23842	tar_cand_23842_60_29	59	29	CD			
		HBII-52_Ts31139	tar_cand_31139_264_79	79	79	CD			
		HBII-52_Ts23821	tar_cand_23821_237_2265	81	2265	CD			
		HBII-52_Ts23839	tar_cand_23839_116_30	48	30	CD			

		HBII-52_Ts23779	tar_cand_23779_53_28	53	28	CD						
		HBII-52_Ts31057	tar_cand_31057_268_50	61	50	CD						
		HBII-52_Ts31051	tar_cand_31051_195_866	81	866	CD						
		HBII-52_Ts32713	tar_cand_32713_144_158	82	158	CD						
		HBII-52_Ts31070	tar_cand_31070_135_2338	82	2338	CD						
		HBII-52_Ts32644	tar_cand_32644_96_1054	81	1054	CD						
		HBII-52_Ts32715	tar_cand_32715_77_20	60	20	CD						
**1**	**2**	**3**	**4**	**5**	**6**	**7**	**8**		**9**		**10**	
	HBII-85-25	HBII-85-25_Ts31247	tar_cand_31247_181_7226	94	7226	CD	?					
		HBII-85-25_Ts31251	tar_cand_31251_255_128024	94	128024	CD	?					
		HBII-85-25_Ts31248	tar_cand_31248_154_201470	94	201470	CD	?					
		HBII-85-25_Ts31249	tar_cand_31249_163_47137	94	47137	CD	?					
	HBII-436	HBII-436_Ts27686	tar_cand_27686_324_20839	72	20839	CD	?					
	HBII-438B	HBII-438B_Ts33982	tar_cand_33982_112_7056	67	7056	CD	?					
SF3B3	HBII-82	HBII-82_Ts26370	tar_cand_26370_133_3001	90	3001	CD	28S	G3923				
	HBII-82B	HBII-82B_Ts26369	tar_cand_26369_100_1973	80	1973	CD	28S	G3923				
NOP5	HBII-95B	HBII-95B_Ts28687	tar_cand_28687_136_51960	87	51960	CD	18S	G509				
		HBII-95B_Ts28686	tar_cand_28686_245_37650	88	37650	CD						
	HBII-234	HBII-234_Ts28689	tar_cand_28689_114_3309	87	3309	CD	18S	A512				
		HBII-234_Ts28690	tar_cand_28690_117_9813	85	9813	CD						

GNL3	HBII-108	HBII-108 _Ts14163	tar_cand_14163_111_12096	76	12096	CD	18S	G683				
	HBII-108B	HBII-108B_Ts14164	tar_cand_14164_205_1935	82	1935	CD	18S	G683				
	HBII-210	HBII-210_Ts14165	tar_cand_14165_174_161947	77	161947	CD	28S	G4464				
GLTSCR2	HBII-115	HBII-115_Ts39790	tar_cand_39790_109_103	109	103	CD	?					
EIF4G1	HBII-142	HBII-142_Ts25470	tar_cand_25470_282_19890	76	19890	CD	18S	C1272				
ch-TOG	HBII-166	HBII-166_Ts28968	tar_cand_28968_163_335179	111	335179	CD	U6	C60				
RPL13	HBII-202	HBII-202_Ts4086	tar_cand_4086_279_4719	71	4719	CD	18S	U428	28S	A2388		
**1**	**2**	**3**	**4**	**5**	**6**	**7**	**8**		**9**		**10**	
AP1G1	HBII-239	HBII-239 _Ts34514	tar_cand_34514_173_6542	86	6542	CD	5.8S	U14				
RPL37	HBII-240	HBII-240_Ts10273	tar_cand_10273_101_1905	83	1905	CD	28S	U4590				
AY264285	HBII-276	HBII-276 _Ts30962	tar_cand_30962_237_21783	76	21783	CD	28S	G3723				
MNAB/OR1K1	HBII-295	HBII-295 _Ts25467	tar_cand_25467_116_91031	97	91031	CD	?					
FLJ10534	HBII-296A	HBII-296A_Ts19449	tar_cand_19449_125_6200	92	6200	CD	28S	G4588				
D26488	HBII-316	HBII-316_Ts16646	tar_cand_16646_136_242459	89	242459	CD	28S	A3846				

CCAR1	HBII-419	HBII-419 _Ts38909	tar_cand_38909_115_128505	68	128505	CD	18S	G867				
EST cluster	snR38A	snR38A _Ts5610	tar_cand_5610_87_503	72	503	CD	28S	G4362				
	snR38B	snR38B _Ts5609	tar_cand_5609_123_6234	88	6234	CD	28S	G4362				
	snR38C	snR38C_Ts5608	tar_cand_5608_130_4197	80	4197	CD	28S	G4362				
n-cod MEG8	14q(I-3)	14q(I-3)_Ts21762	tar_cand_21762_107_92719	71	92719	CD	?					
	14q(I-4)	14q(I-4)_Ts21765	tar_cand_21765_149_75009	75	75009	CD	?					
	14q(I-6)	14q(I-6)_Ts21768	tar_cand_21768_119_106637	74	106637	CD	?					
	14q(I-7)	14q(I-7)_Ts21769	tar_cand_21769_116_68936	76	68936	CD	?					
	14q(I-8)	14q(I-8)_Ts21770	tar_cand_21770_127_143358	73	143358	CD	?					
	14q(I-9)	14q(I-9)_Ts21771	tar_cand_21771_116_104059	71	104059	CD	?					
	14q(II-1)	14q(II-1)_Ts21772	tar_cand_21772_103_31345	71	31345	CD	?					
	14q(II-3)	14q(Ii-3)_Ts21773	tar_cand_21773_100_29443	74	29443	CD	?					
SNRPB	SNORD119	SNORD119_Ts9360	tar_cand_9360_171_6103	82	6103	CD	28S	A4560				
**1**	**2**	**3**	**4**	**5**	**6**	**7**	**8**		**9**		**10**	
UBAP2	SNORD121A	SNORD121A_Ts30747	tar_cand_30747_108_3826	91	3826	CD	28S	G4607				
	SNORD121B	SNORD121B_Ts30748	tar_cand_30748_94_917	73	917	CD	28S	G4607				
THRAP4/MED24	SNORD124	SNORD124_Ts4547	tar_cand_4547_173_2768	108	2768	CD	?					

AP1B1	SNORD125	SNORD125_Ts35282	tar_cand_35282_113_25706	94	25706	CD	?						
CCNB1IP1	SNORD126	SNORD126_Ts23875	tar_cand_23875_110_2122	77	2122	CD	?						
PRPF39	SNORD127	SNORD127_Ts22820	tar_cand_22820_126_12646	87	12646	CD	18S	G1447					
MAGED4	SNORA11D	SNORA11D_Ts9295	tar_cand_9295_190_16655	126	16655	HACA	?						
IARS	SNORA84	SNORA84_Ts3732	tar_cand_3732_197_34476	132	34476	HACA	?						
AB051518	mgU2-19/30/Z32	mgU2-19/30_Ts3876	tar_cand_3876_363_19838	267	19838	SCARNA9	U2	G19	U2	A30			
	mgU2-25/61	mgU2-25/61_Ts3658	tar_cand_3658_241_20843	236	20843	SCARNA2	U2	G25	U2	C61			
AB046784	mgU6-53B	mgU653B_Ts24620	tar_cand_24620_176_93986	109	93986	CD	U6	A53					
		mgU653B_Ts24621	tar_cand_24621_138_531	109	531	CD							
EST cluster	mgU6-47	mgU6-47_Ts40664	tar_cand_40664_148_47024	96	47024	CD	U6	A47					

Supplementary Table 3. Tarsier miRNAs and their tarsier homologs

human miRNA	tarsier miRNA	draft name
hsa-mir-30e	tsy-mir-30e	tar_cand_29159_74_6176
hsa-mir-30c-1	tsy-mir-30c-1	tar_cand_29158_80_12464
hsa-mir-9-1	tsy-mir-9-1	tar_cand_4807_135_28545
hsa-mir-488	tsy-mir-488	tar_cand_10209_60_31
hsa-mir-181a-1	tsy-mir-181a-1	tar_cand_15720_82_40047
hsa-mir-29c	tsy-mir-29c	tar_cand_33118_168_12736
hsa-mir-29b-2	tsy-mir-29b-2	tar_cand_33119_326_16670

hsa-mir-26b	tsy-mir-26b	tar_cand_20298_57_417
hsa-mir-153-1	tsy-mir-153-1	tar_cand_22919_56_326
hsa-mir-149	tsy-mir-149	tar_cand_3840_99_94967
hsa-mir-885	tsy-mir-885	tar_cand_21449_59_4832
hsa-mir-26a-1	tsy-mir-26a-1	tar_cand_33189_94_30382
hsa-mir-425	tsy-mir-425	tar_cand_32909_93_4578
hsa-mir-191	tsy-mir-191	tar_cand_32908_74_65273

hsa-let-7g	tsy-let-7g	tar_cand_12031_212_9767
hsa-mir-16-2	tsy-mir-16-2	tar_cand_12227_158_1124
hsa-mir-218-1	tsy-mir-218-1	tar_cand_12846_153_2965
hsa-mir-1973	tsy-mir-1973	tar_cand_3422_142_136
hsa-mir-874	tsy-mir-874	tar_cand_26579_83_101
hsa-mir-103a-1	tsy-mir-103-1	tar_cand_13781_154_60929
hsa-mir-218-2	tsy-mir-218-2	tar_cand_13793_67_2912

hsa-mir-340	tsy-mir-340	tar_cand_2340_69_998
hsa-mir-339	tsy-mir-339	tar_cand_11845_63_1442
hsa-mir-590	tsy-mir-590	tar_cand_37447_285_1402
hsa-mir-93	tsy-mir-93	tar_cand_4955_67_929
hsa-mir-106b	tsy-mir-106b	tar_cand_4954_106_1317
hsa-mir-335	tsy-mir-335	tar_cand_6925_121_726
hsa-mir-490	tsy-mir-490	tar_cand_13881_111_360

hsa-mir-151a	tsy-mir-151	tar_cand_5778_116_4555
hsa-mir-101-2	tsy-mir-101-2	tar_cand_6827_62_14699
hsa-mir-491	tsy-mir-491	tar_cand_11447_68_612
hsa-mir-7-1	tsy-mir-7-1	tar_cand_2371_131_1040
hsa-let-7a-1	tsy-let-7a-1	tar_cand_21871_223_9892
hsa-let-7f-1	tsy-let-7f-1	tar_cand_21872_126_4842
hsa-mir-628	tsy-mir-628	tar_cand_27738_159_707

hsa-let-7e	tsy-let-7e	tar_cand_37865_77_11397
hsa-mir-125a	tsy-mir-125a	tar_cand_37866_173_76186
hsa-mir-99a	tsy-mir-99a	tar_cand_3997_193_54354
hsa-let-7c	tsy-let-7c	tar_cand_3996_171_2976
hsa-mir-125b-2	tsy-mir-125b-2	tar_cand_3992_111_166833
hsa-mir-185	tsy-mir-185	tar_cand_21490_58_3703
hsa-mir-33a	tsy-mir-33a	tar_cand_24250_61_62

hsa-mir-1249	tsy-mir-1249	tar_cand_14745_185_131
hsa-let-7a-3	tsy-mir-21871	tar-cand-21871-223-9892
hsa_can_1288	tsy_can_1288-1	tar_cand_20618_71_759
	tsy_can_1288-2	tar_cand_7957_152_188455
	tsy_can_1288-3	tar_cand_36874_108_477
	tsy_can_1288-4	tar_cand_22213_71_29
	tsy_can_1288-5	tar_cand_7098_90_229228

	tsy_can_1288-8	tar_cand_18346_58_194
	tsy_can_1288-9	tar_cand_23569_53_226
	tsy_can_1288-11	tar_cand_6695_71_188999
hsa-let-7d	tsy-let-7d	tar_cand_21873_296_8694
hsa-mir-455	tsy-mir-455	tar_cand_18483_63_2757
hsa-mir-126	tsy-mir-126	tar_cand_39833_110_9715
hsa-mir-221	tsy-mir-221	tar_cand_37624_82_2376

		tar_cand_37625_87_3626
hsa-mir-98	tsy-mir-98	tar_cand_7333_82_1015
hsa-let-7f-2	tsy-let-7f-2	tar_cand_7332_94_5012
hsa-mir-545	tsy-mir-545	tar_cand_15746_168_32
hsa-mir-361	tsy-mir-361	tar_cand_23549_97_821
hsa-mir-1264	tsy-mir-1264	tar_cand_10622_64_159
hsa-mir-1298	tsy-mir-1298	tar_cand_10625_74_44

hsa-mir-448	tsy-mir-448	tar_cand_25859_78_210
hsa-mir-767	tsy-mir-767	tar_cand_6513_57_39
hsa-mir-1296	tsy-mir-1296	tar_cand_16713_68_315
hsa-mir-346	tsy-mir-346	tar_cand_8702_62_218
hsa-mir-107	tsy-mir-107	tar_cand_7350_77_21210
hsa_can_1424	tsy_can_1424	tar_cand_29273_65_75
hsa-mir-192	tsy-mir-192	tar_cand_7213_63_777

hsa-mir-139	tsy-mir-139	tar_cand_35112_84_7168
hsa-mir-125b-1	tsy-mir-125b-1	tar_cand_16250_192_166649
hsa-let-7a-2	tsy-let-7a-2	tar_cand_16274_87_9539
hsa-mir-148b	tsy-mir-148b	tar_cand_2497_154_296
hsa-mir-26a-2	tsy-mir-26a-2	tar_cand_3694_238_30219
hsa-mir-331	tsy-mir-331	tar_cand_22186_365_980
hsa-mir-16-1	tsy-mir-16-1	tar_cand_25714_274_14341

hsa-mir-342	tsy-mir-342	tar_cand_7325_71_29529
hsa_can_1288	tsy-can_1288	tar_cand_16678_120_3379
hsa-mir-326	tsy-mir-326	tar_cand_9690_211_895
mir-100	tsy-mir-100	tar_cand_16276_185_8669
MIR25	tsy-MIR25	tar_cand_4956_60_129
MIR488	tsy-MIR488	tar_cand_10170_60_31
MIR504	tsy-MIR504	tar_cand_9008_57_266

Mir350	tsy-Mir350	tar_cand_33923_64_91
MIR764	tsy-MIR764	tar_cand_10620_63_123
MIR652	tsy-MIR652	tar_cand_25568_52_1257

Supplementary Table 4. A summary of repeat elements specific to the tarsier genome

	Families	Family number	Copy number x1000	Full length copies	Total Length (Mb)	% of Genome	Tarsus specific x1000	Active families
	**LINEs**	**148**	**981,73**	**1,02**	**666,47**	**19,57%**	**54,39**	**9**
L1	L1M	84	442,64	0,058	263,85	7,75%		
	L1P	32	49,47	0,003	29,35	0,86%	4,70	2
	L1_TS	7	342,70	0,929	336,74	9,89%	49,69	7
	LINE2	4	124,68	-	31,65	0,93%		
	LINE3/CR1	15	14,63	0,028	3,03	0,09%		
	Other	6	7,61	0,002	1,85	0,05%		
	**SINEs**	**16**	**1.696,70**	**936,87**	**410,15**	**12,04%**	**175,40**	**5**
Alu	Alu-TS	3	1.257,80	855,008	337,91	9,92%	168,10	3
	AluJ	2	142,80	48,119	31,53	0,93%		
	Monomers	3	56,73	25,886	7,19	0,21%	7,30	2
MIR	MIRm	2	210,88	7,399	30,45	0,89%		
	MIR3	1	26,56	0,411	2,82	0,08%		
	Other	5	1,93	0,044	0,25	0,01%		
	**TINE-pseudogenes**	**3**	**30,49**	**28,496**	**2,38**	**0,07%**	**4,66**	**2**
	**LTR-retroposons**	**260**	**447,39**	**0,088**	**197,04**	**5,79%**	**5,88**	**12**
	ERV class I	140	110,68	0,029	64,57	1,90%	3,52	4
	ERV(K) class II	19	34,84	0,016	23,51	0,69%	1,15	4
	ERV(L) class III	54	104,57	0,012	44,23	1,30%	0,30	2
	MaLR	37	189,14	0,031	62,86	1,85%	0,91	2
	Other	10	8,16	-	1,87	0,05%		
	**DNA-transposons**	**219**	**368,35**	**69,258**	**89,52**	**2,63%**	**1,08**	**8**
	MuDR	5	1,72	0,027	0,66	0,02%		
	Piggi-Bac	4	1,33	0,017	0,31	0,01%		
hAT	hAT	6	7,22	2,000	1,01	0,03%		
	BlackJack	8	13,91	4,246	2,64	0,08%		
	Charlie	70	190,38	42,124	37,49	1,10%	0,76	3
	Tip100-Zaphod	30	24,51	2,457	5,48	0,16%		
Tc1	TcMar	3	0,98	0,019	0,12	0,00%		
	Tc2	1	0,98	0,003	0,28	0,01%		

Pogo	1	0,03	0,005	0,01	0,00%		
Tigger	51	86,82	16,185	32,15	0,94%	0,20	3
Mariner	5	36,48	1,825	8,75	0,26%	0,12	2
RC/Helitron	4	1,31	0,019	0,27	0,01%		
Other	31	2,68	0,331	0,35	0,01%		
**small RNA pseudogenes**	**65**	**12,99**	**4,421**	**1,25**	**0,03%**	**1,72**	
tRNA	47	1,63	0,896	0,09	0,00%		
U-RNA	11	4,09	2,274	0,38	0,01%	0,82	
Other	7	7,27	1,251	0,78	0,02%	0,90	
**Unclassified**	**29**	**1,85**	**0,013**	**0,296**	**0,01%**		
**Sattelite**				**42,332**	**1,24%**		
**Simple/Low complecity**				**83,972**	**2,47%**		
**Sattelite, Simple, Low complecity**				**126,305**	**3,71%**		
**Total**	**740**	**3.539,50**	**1.040,16**	**1.493,41**	**43,85%**	**243,13**	**36**

Supplementary Table 5. Tarsier scaffold sequence coordinates for the numt positions for various species

**Primate specific numt location**
scaffold_5015 18357-18535

**Mouse lemur specific numt scaffold coordinates**
scaffold_1807 139276-139448
scaffold_3153 82365-82554
scaffold_343 85814-86010
scaffold_410 208267-208442
scaffold_79 718054-718237

Supplementary Table 6. Comparative numt positions of primate species used to verify tarsier numt locations

**Human numt coordinates**	**Numts**	**Locations**
chr2:33992538-33992593	HSA_NumtS_050_b1	5'-UTR
chr3:25508995-25509033	HSA_NumtS_133_b1	intron/5'-UTR
chr5:73071717-73071757	HSA_NumtS_214_b1	intron
chr20:13147959-13148001	HSA_NumtS_544_b1	intergenic
chr22:36281719-36281765	HSA_NumtS_560_b1	intron/5'-UTR
chr13:110076472-110076727	HSA_NumtS_472_b1	intergenic
chr1:38077348-38077421	HSA_NumtS_009_b1	3'-UTR
chr5:165957424-165957466	HSA_NumtS_232_b1	intergenic
chr8:100508098-100508181	HSA_NumtS_319_b1	intron
chr4:47774289-47774381	HSA_NumtS_179_b1	3'-UTR/intron/5'-UTR
chr11:73221706-73221868	HSA_NumtS_398_b1	5'-UTR
chr11:122874314-122874385	HSA_NumtS_410_b1	intergenic
chr12:41757437-41757525	HSA_NumtS_426_b1	intron
chr20:9149571-9149612	HSA_NumtS_543_b1	5'-UTR
chr20:55639110-55639179	HSA_NumtS_546_b1	intergenic

chr2:81893601-81893852	HSA_NumtS_058_b1	3'-UTR
chr7:145694426-145694521	HSA_NumtS_289_b1	intergenic
chr18:45379617-45379808	HSA_NumtS_522_b1	3'-UTR/intron
chr17:51183094-51183746	HSA_NumtS_513_b1	intergenic
chr4:56194327-56194457	HSA_NumtS_182_b1	intergenic
chr13:56545768-56545893	HSA_NumtS_464_b1	intergenic
chr17:42075084-42075151	HSA_NumtS_512_b1	5'-UTR

**Rheus macaque numt coordinates**	**Locations**
chr1:215872927-215873137	intergenic
chr11:130692898-130693035	intergenic
chr12:81781081-81781311	intergenic
chr14:90613401-90613633	intergenic
chr19:49842963-49843088	intergenic
chr20:707323-707682	intron
chr20:22031226-22031350	intergenic
chr20:75142863-75143048	intergenic
chr3:119563855-119564027	intergenic
chr3:91233920-91234290	intergenic
chr5:64025193-64025548	intergenic
chr7:59771551-59771682	intergenic
chr8:100168419-100168678	intergenic
chr9:67885501-67885637	intergenic
chr9:114026008-114026145	intergenic
chrX:65478830-65478988	intergenic

**Marmoset numt coordinates**	**Locations**
chr1:199813559-199814107	intergenic
chr11:129455234-129455860	intergenic
chr12:100355784-100355905	intergenic

chr16:73532845-73532996	intergenic
chr16:74005431-74005618	intergenic
chr18:28397992-28398324	intergenic
chr20:37203021-37203271	intergenic
chr4:30482948-30483064	intergenic
chr4:69887632-69887777	intergenic
chr5:105755984-105756252	intergenic
chr7:4760158-4760443	intergenic
chr7:37947789-37947942	intergenic
chr8:49043875-49044101	intergenic
chr8:118531084-118531601	intergenic

Supplementary Table 7. Tarsier genes under positive selection for branch site tests

ALKBH1	GCHFR	LOC103276389	SGCG
ANKRD16	GPR26	LOH12CR1	SHC4
APEH	GRXCR2	LRR1	SHISA2
APOBR	GSX1	LRRC71	SHISA3
ARL10	HINT3	LRWD1	SKAP1
ATG10	HSD17B14	LYPD4	SLC14A2
ATP6V1E2	HTR6	LYZ	SLC35F6
BBS2	ICAM3	MAB21L3	SLC51B
BFSP2	IDUA	MCL1	SLC7A9
BPIFA2	IL18R1	MRPL51	SNX15
C15orf26	IL1A	MTAP	SOGA3
CACNG2	IL22RA1	MURC	SPATA24
CBX7	IMPG1	MVD	SRD5A3
CCDC115	IPPK	MZT1	STXBP3
CCDC68	KCTD2	NEIL3	TAC1
CCDC70	KHDRBS2	NEU2	TBKBP1
CD248	KIAA0101	NGB	TCAP

CHP2	KLF7	NPPC	TCP11L2
CLVS2	LAMTOR2	NSMCE2	TIMM50
CNIH2	LHFPL2	NUBP2	TMC8
CNTRL	LOC103249508	OAF	TMEM109
CPA6	LOC103249803	OTUB2	TMEM154
CRHBP	LOC103250458	PDGFB	TMEM179B
CRYL1	LOC103250912	PDLIM3	TMEM182
DDX20	LOC103252421	PFN2	TMEM70
DECR2	LOC103254319	PIFO	TMPRSS12
DKK4	LOC103256425	PLAC9	TREM1
DLX2	LOC103257868	PMCH	TRMT11
DNAJC5B	LOC103259314	PNPLA4	TSHB
DNAL1	LOC103261001	POLD4	TTC38
DNPH1	LOC103261002	PPAT	TTF2
DTD1	LOC103261647	PRL	TYW3
DTD2	LOC103262295	PSMB1	UBE2S
EIF3G	LOC103262757	PTPLAD2	UPRT
EIF4EBP1	LOC103263294	PTRH1	UTP11L
ELF3	LOC103263575	PTS	VASH1
F13B	LOC103263722	PTTG1IP	VSTM2L
FAM185A	LOC103264564	PTX3	WDR77
FAM216B	LOC103264591	PYURF	WDYHV1
FAM229B	LOC103265837	RASL11A	WNT11
FGF12	LOC103266202	RBM43	WTIP
FICD	LOC103266491	REM1	YIF1A
FITM2	LOC103266568	RFTN2	YIPF4
GABRQ	LOC103267768	RGCC	ZNF18
GADD34	LOC103269326	RGR	ZNF366
GALNT7	LOC103272308	RNF139	ZNF518B
GAPT	LOC103273909	RNF5	ZNF704
GAS2L2	LOC103276387	RTP3	ZP2

Supplementary Table 8. Diseases detected for genes deemed under positive selection within tarsier

Disease	Impact	Source	Gene(s)	P-value	q-value FDR B&H
Factor XIII Deficiency	cardiovascular	CTD	F13B	0.014	0.040
Infarction	cardiovascular	CTD	PRL	0.007	0.027
Hypotension	cardiovascular	CTD	TAC1, IL1A,PRL	0.004	0.027
Bradycardia	cardiovascular	CTD	TAC1,PRL	0.004	0.027
Dilated cardiomyopathy 1N	cardiovascular	Clinical Variations	TCAP	0.007	0.027
Primary familial hypertrophic cardiomyopathy	cardiovascular	Clinical Variations	TCAP	0.014	0.040
Galactorrhea	endocrine (lactation)	CTD	PRL	0.007	0.027
Gynecomastia	endocrine (lactation)	CTD	PRL	0.014	0.040
Puerperal Disorders	endocrine (thyroid)	CTD	PRL	0.007	0.027
Hypothyroidism, Congenital, Nongoitrous, 4	endocrine (thyroid)	CTD	TSHB	0.007	0.027
Gingival Hyperplasia	gums/mouth	CTD	PDGFB	0.014	0.040
Deafness, autosomal recessive 101	hearing	Clinical Variations	GRXCR2	0.007	0.027
Chorioamnionitis	immune	CTD	IL1A	0.007	0.027
Fever	immune	CTD	IL1A,PRL	0.008	0.032
Lymphoma, T-Cell, Cutaneous	immune	CTD	MCL1	0.014	0.040
Multiple Myeloma	immune	CTD	MCL1,CBX7	0.016	0.046
Cystinuria	kidney	OMIM	SLC7A9	0.014	0.040
Hurler syndrome	lysosomal (musculoskeletal and visual impacts)	OMIM	IDUA	0.007	0.027
Hurler-Scheie Syndrome	lysosomal (musculoskeletal and visual impacts)	OMIM	IDUA	0.007	0.027
Mucopolysaccharidosis I	lysosomal (musculoskeletal and visual impacts)	CTD	IDUA	0.007	0.027
Scheie Syndrome	lysosomal (musculoskeletal and visual impacts)	OMIM	IDUA	0.007	0.027
Immunodeficiency due to defect in mapbp-interacting protein	musculoskeletal	Clinical Variations	LAMTOR2	0.007	0.027

Disease	Impact	Source	Gene(s)	P-value	q-value FDR B&H
Diaphyseal medullary stenosis with malignant fibrous histiocytoma	musculoskeletal	CTD	MTAP	0.007	0.027
Myopathy, limb-girdle, with bone fragility	musculoskeletal	CTD	MTAP	0.007	0.027
Limb-girdle muscular dystrophy, type 2C	musculoskeletal	CTD	SGCG	0.007	0.027
Mental retardation, autosomal dominant 10	neurological	Clinical Variations	CACNG2	0.007	0.027
Epilepsy, familial temporal lobe, 5	neurological	Clinical Variations	CPA6	0.007	0.027
Febrile seizures, familial, 11	neurological	Clinical Variations	CPA6	0.007	0.027
Idiopathic basal ganglia calcification 5	neurological	Clinical Variations	PDGFB	0.007	0.027
Phobic Disorders	neurological	CTD	PRL	0.014	0.040
Sexual Dysfunction, Physiological	neurological	CTD	PRL	0.007	0.027
6-pyruvoyl-tetrahydropterin synthase deficiency	neurological	CTD	PTS	0.007	0.027
Hyperphenylalaninemia BH4-Deficient, A HPABH4A	neurological	OMIM	PTS	0.007	0.027
Sialorrhea	neurological	CTD	TAC1	0.007	0.027
Kearns-Sayre Syndrome	neurological (musculoskeletal and visual impacts)	CTD	IL1A	0.014	0.040
MERRF Syndrome	neurological (musculoskeletal and visual impacts)	CTD	IL1A	0.014	0.040
Kahrizi syndrome	neurological (musculoskeletal and visual impacts)	Clinical Variations	SRD5A3	0.007	0.027
Nuclearly-encoded mitochondrial complex V (ATP synthase) deficiency 2	neuromuscular	Clinical Variations	TMEM70	0.007	0.027
Ciliary dyskinesia, primary, 16	respiratory	Clinical Variations	DNAL1	0.007	0.027
Cutaneous nevi	skin	GWAS	MTAP	0.014	0.040
Dermatofibrosarcoma protuberans	skin	Clinical Variations	PDGFB	0.007	0.027
Epidermodysplasia Verruciformis	skin	CTD	TMC8	0.014	0.040
Bardet-Biedl syndrome 2	vision	Clinical Variations	BBS2	0.007	0.027

Disease	Impact	Source	Gene(s)	P-value	q-value FDR B&H
Cataract, Autosomal Dominant, Multiple Types 1	vision	OMIM	BFSP2	0.007	0.027
Macular dystrophy, vitelliform, 4	vision	Clinical Variations	IMPG1	0.007	0.027
Retinitis pigmentosa 44	vision	Clinical Variations	RGR	0.007	0.027
Congenital disorder of glycosylation type 1Q	vision	Clinical Variations	SRD5A3	0.007	0.027

Supplementary Table 9. Tarsier orthologous gene sequences to human tested for significant associations with curated pathway databases

**Pathway Name**	**Source**	**Genes**	**Pathway Count**	**Observed Genes**	**Expected**	**Ratio of Enrichment**	**Raw P-Value**	**Adjusted P-Value**
Metabolic pathways	KEGG	ATP6V1E2 CRYL1 GALNT7 IDUA IPPK MTAP MVD POLD4 PPAT PTS UPRT	1130	11	4.14	2.66	0.0031	0.0267
MAPK signaling pathway	KEGG	CACNG2 CHP2 FGF12 IL1A PDGFB	268	5	0.98	5.09	0.0032	0.0267
Cytokine-cytokine receptor interaction	KEGG	IL18R1 IL1A IL22RA1 PDGFB PRL	265	5	0.97	5.15	0.003	0.0267
Base excision repair	KEGG	NEIL3 POLD4	33	2	0.12	16.54	0.0065	0.0406
Hypertrophy Model	WikiPathways	IL1A EIF4EBP1	20	2	0.07	27.3	0.0024	0.0288
Lymphocyte TarBase	WikiPathways	ARL10 GALNT7 MCL1 PTRH1 SNX15 TMEM109 UBE2S	533	7	1.95	3.58	0.0036	0.0288
negative regulation of fibroblast growth factor production	GO Biological Process	RGCC WNT11	2	2	0.02	117.98	7.13E-05	0.0357
positive regulation of mitosis	GO Cellular Component	IL1A NSMCE2 PDGFB RGCC	36	4	0.31	13.11	0.0002	0.0357

Supplementary Table 10. Species included in the analysis of positive selection

Species		Version	Source	Date
Homo sapiens	hsa	hg19	CCDS/UCSC	download 30.07.2014
Pan troglodytes	ptr	2.1.4.75	Ensembl	Nov 2012
Gorilla gorilla	ggo	3.1.75	Ensembl	Jul 2011
Macaca mulatta	mac	1.75	Ensembl	May 2010
Callithrix jacchus	cja	3.2.1.75	Ensembl	Feb 2014
Tarsius syrichta	tsy	2.0.1	NCBI	Sep 2013
Otolemur garnettii	oga	3.75	Ensembl	Dec 2011
Tupaia belangeri	tbe	1.75	Ensembl	Apr 2013

Supplementary Methods

RepeatMasker parameter settings. For the numt insertional analysis, we used the Philippine tarsier mitochondrial genome (NC_012774.1) with a user-defined library sequence (-lib): -e crossmatch –s –nolow –norna –lib. For RepeatMasker, the following parameters were used: –e crossmatch –species primates; settings for npcRNAs: -e crossmatch -lib; library (lib) containing predicted human micro and snoRNAs.

Pairwise alignments. The pairwise alignment (*Homo sapiens*/*Tarsius syrichta*-2.0.1) was based on the soft-masked human genome:
(hg19; http://www.repeatmasker.org/species/hg.html) and was generated as previously described at: (http://genomewiki.ucsc.edu/index.php/Whole_genome_alignment_howto) with the software provided by UCSC (http://hgdownload.cse.ucsc.edu/admin/exe/) in several steps.

Multi-way alignments. The following steps were followed to align whole genome assemblies of targeted primates:

(1) Alignment with LASTZ for each fasta-file of tarsier and human

    lastz target.fasta query.fasta (result: *.lav-format file)

    (target.fasta = tarsier genome; query.fasta = human genome)

(2) converting fasta into a 2bit-format

    faToTwoBit *.fasta (result: target.2bit, query.2bit)

(3) converting the *.lav-formate file into psl-format

    lavToPSL for i in `ls *.lav`; do lavToPsl $i ${i%.lav}.psl; done

(4) chaining

    a) axtChain

        for i in `ls *.psl`; do axtChain $i target.2bit query.2bit –scoreScheme='file-name' –minScore=5000 –linearGap=medium –psl ${i%.psl}.chain

    b) faSize (determining the file sizes of the fasta-files using a python script)

    c) chainMergeSort (to merge and sort all chain-files into one file; default settings)

        chainMergeSort *.chain > all.chain

    d) chainPreNet (to filter the chain-file)

        chainPreNet all.chain target.sizes query.sizes all.pre.chain

    e) chain joining occurred under these threshold: axtChain; parameters

        −chainMinScore = 3000 −chainLinearGap = medium

(5) netting

    a) chainNet (combining chains into nets)

chainNet all.pre.chain target.sizes query.sizes all_target.net all_query.net

b) netToAxt (to convert the net-format into the readable axt-format

netToAxt all_target.net all.pre.chain target.2bit query.2bit out.axt

The *Tarsius syrichta* genome (Version 2.0.1) along with the genomes of human (*Homo sapiens*; Dec. 2013; GRCh38/hg38), chimpanzee (*Pan troglodytes*; Feb. 2011; CSAC 2.1.4/panTro4), bonobo (*Pan paniscus*; May 2012; Max-Planck/panPan1), gorilla (*Gorilla gorilla*; May 2011; gorGor3.1/gorGor3), orangutan (*Pongo pygmaeus abelii*; Jul. 2007; WUGSC 2.0.2/ponAbe2), gibbon (*Nomascus leucogenys*; Oct. 2012; GGSC Nleu3.0/nomLeu3), rhesus macaque (*Macaca mulatta*; Oct. 2010; BGI CR_1.0/rheMac3), crab-eating macaque (*Macaca fascicularis*; Jun. 2013; Macaca_fascicularis_5.0/macFas5), baboon (*Papio anubis*; Mar. 2012; Baylor Panu_2.0/papAnu2), green (vervet) monkey (*Chlorocebus sabaeus*; Mar. 2014; Chlorocebus_sabeus 1.1/chlSab2), proboscis monkey (*Nasalis larvatus*; Nov. 2014; Charlie1.0/nasLar1), golden snubnosed monkey (*Rhinopithecus roxellana*; Oct. 2014; Rrox_v1/rhiRox1), marmoset (*Callithrix jacchus*; Mar. 2009; WUGSC 3.2/calJac3), squirrel monkey (*Saimiri boliviensis*; Oct. 2011; Broad/saiBol1), mouse lemur (*Microcebus murinus*; Jul. 2007; Broad/micMur1), bushbaby (*Otolemur garnettii*; Mar. 2011; Broad/otoGar3), and tree shrew (*Tupaia belangeri*; Dec. 2006; Broad/tupBel1), mouse (*Mus musculus*; Dec. 2011; GRCm38/mm10) and dog (*Canis lupus familiaris*; Seq. 2011; Broad/canFam3) as outgroups were used in this alignment. The 20-way alignment is implemented in GPAC[1] and available at: http://hgdownload.soe.ucsc.edu/goldenPath/tarSyr2/multiz20way/.

Positive gene selection. For human, high-quality consensus CDS (CCDS) data were downloaded from the UCSC Table Browser[2] and translated into protein sequences using *transeq*[3] (settings: transeq –sequence ccds –frame 1). For the tarsier, we used the protein sequences and the rna.asn-file from: ftp://ftp.ncbi.nlm.nih.gov/genomes/Tarsius_syrichta/ to retrieve the CDS sequences through the use of a script (asn2all; settings: ./mac.asn2all -fd -o CDS_from_transcripts.fsa -i rna.asn; NCBI C++ toolkit). To identify orthology groups we used orthoMCL[4]: *orthomclAdjustFasta, orthomclFilterFasta* with min_length=10; max_percent_stops=20, all versus all blast of all good proteins (defined by *orthomclFilterFasta*) with *blastall* -m 8 -z 218855 -F 'm S' -v 100000 -b 100000 -e 1e-5, *orthomclBlastParser.*

Pairwise Sequentially Markovian Coalescent (PSMC). In SAMtools[5] we built the diploid.fq.gz file by applying the following command:

1) samtools mpileup -C50 -uf T_syrichta.fa T_syrichta.bam| bcftools call -c - \
|vcfutils.pl vcf2fq -d 10 -D 100 | gzip > diploid.fq.gz (for parameters, see SAMtools description https://github.com/samtools/samtools)

In a local version of PSMC we ran the following commands (for parameters, see PSMC description https://github.com/lh3/psmc):
2) utils/fq2psmcfa -q20 diploid.fq.gz > diploid.psmcfa
3) psmc -N25 -t15 -r5 -p "4+25*2+4+6" -o diploid.psmc diploid.psmcfa
4) utils/psmc2history.pl diploid.psmc | utils/history2ms.pl > ms-cmd.sh
5) utils/psmc_plot.pl -g7 -u1.1e-08 diploid diploid.psmc
for "seven" years per generation (IUCN http://www.iucnredlist.org/details/21492/0) and the human mutation rate 1.1e-08 [6,7].

To complete 100 rounds of bootstrapping, we split the long sequences into shorter fragments and randomly sampled them with replacement in PSMC:
6) ./utils/splitfa diploid.psmcfa > split.psmcfa
7) psmc -N25 -t15 -r5 -p "4+25*2+4+6" -o diploid.psmc diploid.psmcfa
8) seq 100 | xargs -I "{}" echo psmc -N25 -t15 -r5 -b -p "4+25*2+4+6" -o "round-{}.psmc" split.psmcfa | sh
9) cat diploid.psmc round-*.psmc > combined.psmc
10) utils/psmc_plot.pl -g7 -u1.1e-8 combined combined.psmc
(for MacOSX we replaced xargs –i with xargs –I "{}")

To parallelize the PSMC run of bootstrap data, we replaced step **8)** with:
8) parallel -j10 "psmc -N25 -t15 -r5 -b -p "4+25*2+4+6" -o round-{}.psmc split.psmcfa" :::: <(seq 100)
(GNU parallel; ten processes run in parallel)

Numt-screening.

Primate specific numts: It is not possible to detect species-specific numts solely by using the 46- and 20-way alignments (human as leading species) except for human. Therefore, we developed an approach based on the mouse lemur (micMur1)/mouse (mm10) pairwise alignment (micMur1.mm10.net.axt; http://hgdownload.soe.ucsc.edu/goldenPath/micMur1/vsMm10/micMur1.mm10.net.axt.gz). We screened this alignment for mouse lemur specific insertions greater or equal than 100 nt and allowed maximally 20 nt of mouse sequence within the corresponding region. With this approach, we detected 65,182 mouse lemur specific regions (gaps in mouse). These unique mouse lemur sequences were compared to RepeatMasker results (greater or equal than 100 nt) based on the mouse lemur genome (micMur1; http://hgdownload.soe.ucsc.edu/goldenPath/micMur1/bigZips/micMur1.fa.gz) as target and a dimeric (merging two mouse lemur mitochondrial genomes) version of the mouse lemur mtDNA (KR911907.1) as library (3,161 hits). Because mtDNA is a circular molecule, it is possible that numts can span the end and start region of the linear fasta sequence. Therefore, we used a dimeric mtDNA sequence as library. We allowed up to 100 nt non-mitochondrial flanking sequences in the mouse lemur specific sequence region at the 5'- and 3'-end. All hits were manually checked for orthologous regions in tarsier, marmoset, and gorilla using the UCSC Genome Browser[8]. Using the micMur1/mm10 pairwise alignment, we detected nine potential mouse lemur specific numts. By manual comparison to orthologous hits in tariser, marmoset, and gorilla (UCSC Genome Browser), we confirmed one match as primate specific.

Mouse lemur specific numts: We used the mouse lemur (micMur1)/mouse (mm10) pairwise alignment (micMur1.mm10.net.axt; http://hgdownload.soe.ucsc.edu/goldenPath/micMur1/vsMm10/micMur1.mm10.net.axt.gz) to screen for inserts in mouse lemur equal or greater than 100 nt with not more than 20 nt orthologous mouse sequences (65,182). RepeatMasker was used to search these loci for mitochondrial sequences (micMur1 KR911907.1) (see above), revealing nine matches. These cases where reviewed using the UCSC Genome Browser (see above).

Species-specific numts for human, rhesus macaque, and marmoset: Screenings for human, rhesus macaque, and marmoset specific numts were performed using GPAC [1]. For human, we used the 46-way alignment, whereas for the remaining primate species we used different pairwise alignments (rhesus: rheMac3/hg19, http://hgdownload.soe.ucsc.edu/goldenPath/rheMac3/vsHg19/rheMac3.hg19.net.axt.gz; marmoset: calJac3/hg19, http://hgdownload.soe.ucsc.edu/goldenPath/calJac3/vsHg19/calJac3.hg19.net.axt.gz). Coordinates of human numts were downloaded from the UCSC Table Browser (http://genome.ucsc.edu/cgi-bin/hgTables?command=start; genome: Human, assembly: hg19, group: All Tracks, track: NumtS Sequence), whereas the numts coordinates of rhesus (rheMac3;

http://hgdownload.soe.ucsc.edu/goldenPath/rheMac3/bigZips/rheMac3.fa.gz) and marmoset (caljac3, http://hgdownload.soe.ucsc.edu/goldenPath/calJac3/bigZips/calJac3.fa.gz) were detected via RepeatMasker. The genomes of the rhesus macaque and marmoset were used as targets, and each two concatenated species-specific mtDNA genomes (rhesus: NC_005943.1, marmoset: NC_025586.1) as library sequences (see above) for a RepeatMasker run. Numts equal to or greater than 100 nt were used for further investigations (rhesus 779; marmoset 573). Each species-specific numt match with a clear GPAC presence/absence pattern was further confirmed in the UCSC Genome Browser for presence/absence in other primates. For that, we again used the 46-way alignment information in the case of human. The Chain/Net-Information (Comparative Genomics) of gorilla, orangutan, human, marmoset, tarsier, and mouse was used in the case of the rhesus macaque and the 14-way alignment (with marmoset as leading species) in the case of marmoset. The search for species-specific numts of human, rhesus macaque, and marmoset revealed 22, 16, and 14 matches, respectively (Supplementary Table 3).

Internode numts: For detecting internode numts on the lineage to human (internodes: Strepsirrhini-Tarsiiformes; Tarsiiformes-Anthropoidea; Platyrrhini-Cercopithecoidea; Cercopithecoidea-Hominoidea), we used GPAC[1] and the 46-way alignment with human as leading species as database. For GPAC coordinates, we used the 766 numts listed from the UCSC Table Browser[2]. The GPAC analysis was performed using all primate species and tree shrew as an outgroup. Only clear presence/absence boundaries (+/-) were considered for further analyses. All potential numt insertion events were checked manually with the UCSC Genome Browser (see above).

Analysis of introns and intergenic regions. A total of 49,377,710 cDNAs were aligned to ~30,000 tarsier scaffolds to identify and annotate the entire small non protein-coding RNA (npcRNA) transcriptome. Of these, 45,749,647 mapped to the genome reference and were used for downstream analysis. For enrichment of small npcRNAs, we focused on genomic regions located outside known exons. Local extensions of introns and intergenic intervals were calculated based on the NCBI annotation file (ref_Tarsius_syrichta_2.0.1_top_level.gff3). Intron sizes were minimized to exclude ambiguous cDNA assignments. Individual "samtools view" calls generated SAM output files to generate candidate contigs [5]. Based on cut-off values, specific regions (i.e., intron or intergenic sequences) were selected for downstream analysis and all introns or intergenic intervals that harbored at least twenty cDNAs were further processed. All computations were conducted using custom-designed S-Plus scripts.

In addition to the identification of known human npcRNAs via pairwise alignments, we utilized the number of cDNAs per assembled contig and the candidate length to differentiate background from reliably longer and more frequent candidates. We filtered the entire candidate dataset for RNAs >50 nt. All enrichments were computed based on simple gawk (https://www.gnu.org/software/gawk/) scripts. We also applied filters to remove cDNAs, which related to expressed mRNAs or incomplete processing intermediates of hnRNA (heteronuclear RNA) precursors. Finally, we selected 1,338 potential npcRNAs for downstream analysis.

Supplementary References

1 Noll, A. *et al.* GPAC - Genome presence/absence compiler: A web application to comparatively visualize multiple genome-level changes. *Mol. Biol. Evol.* **32**, 275-286, doi:papers3://publication/doi/10.1093/molbev/msu276 (2015).

2 Karolchik, D. *et al.* The UCSC Table Browser data retrieval tool. *Nucleic Acids Res.* **32**, D493-D496, doi:papers3://publication/doi/10.1093/nar/gkh103 (2004).

3 Rice, P., Longden, I. & Bleasby, A. EMBOSS: the European Molecular Biology Open Software Suite. *Trends Genet.* **16**, 276-277, doi:papers3://publication/doi/10.1016/S0168-9525(00)02024-2 (2000).

4 Li, L., Stoeckert, C. J. & Roos, D. S. OrthoMCL: Identification of ortholog groups for eukaryotic genomes. *Genome Res.* **13**, 2178-2189, doi:papers2://publication/doi/10.1101/gr.1224503 (2003).

5 Li, H. *et al.* The Sequence Alignment/Map format and SAMtools. *Bioinformatics* **25**, 2078-2079, doi:papers2://publication/doi/10.1093/bioinformatics/btp352 (2009).

6 Li, H. & Durbin, R. Inference of human population history from individual whole-genome sequences. *Nature* **475**, 493-496, doi:10.1038/nature10231 (2011).

7 Roach, J. C. *et al.* Analysis of genetic inheritance in a family quartet by whole-genome sequencing. *Science* **328**, 636-639, doi:papers3://publication/doi/10.1126/science.1186802 (2010).

8 Kent, W. J. *et al.* The human genome browser at UCSC. *Genome Res.* **12**, 996-1006, doi:papers3://publication/doi/10.1101/gr.229102 (2002).

9 Friedländer, M. R. *et al.* Evidence for the biogenesis of more than 1,000 novel human microRNAs. *Genome Biol.* **15**, R57-R57, doi:papers3://publication/doi/10.1186/gb-2014-15-4-r57 (2014).

10 Lestrade, L. & Weber, M. J. snoRNA-LBME-db, a comprehensive database of human H/ACA and C/D box snoRNAs. *Nucleic Acids Res* **34**, D158-162, doi:10.1093/nar/gkj002 (2006).

11 Cavaille, J. *et al.* Identification of brain-specific and imprinted small nucleolar RNA genes exhibiting an unusual genomic organization. *Proc Natl Acad Sci U S A* **97**, 14311-14316 (2000).

12 Skryabin, B. V. *et al.* Deletion of the MBII-85 snoRNA gene cluster in mice results in postnatal growth retardation. *PLoS Genetics* **3**, e235-e235, doi:papers3://publication/doi/10.1371/journal.pgen.0030235 (2007).

13 Ender, C. *et al.* A human snoRNA with microRNA-like functions. *Molecular cell* **32**, 519-528 (2008).

14 Kehr, S., Bartschat, S., Tafer, H., Stadler, P. F. & Hertel, J. Matching of soulmates: Coevolution of snoRNAs and their targets. *Mol. Biol. Evol.* **31**, 455-467, doi:papers3://publication/doi/10.1093/molbev/mst209 (2014).
